# Some inverse problems in particle physics

**DOI:** 10.1140/epjs/s11734-026-02466-w

**Published:** 2026-07-27

**Authors:** Luigi Del Debbio

**Affiliations:** School of Physics and Astronomy, The University of Edinburgh, Peter Guthrie Tait Road, Edinburgh, EH9 3FD UK

## Abstract

Inverse problems play a central role in current areas of research in particle phenomenology. In these lectures, we focus on two examples, the extraction of Parton Distribution Functions (PDFs) from experimental data (or, equivalently, from pseudo- and quasi-PDFs computed in lattice QCD), and the extraction of spectral functions from lattice Euclidean time correlators. We investigate in detail three different approaches, namely Backus–Gilbert, Gaussian Processes, and fits based on Neural Network parametrizations.

## Introduction

Inverse problems appear in many areas of physics and, more widely, of computational science. The solution of these problems requires a mathematically robust framework that allows the extraction of information about theoretical models from (increasingly large) finite sets of data. It is often the case that solutions are plagued by the ill-posed nature of the problem and require some form of regularization.

In these lectures, we focus on applications to particle phenomenology, a subject area that is increasingly driven toward precision physics as a way to search for new physics beyond the Standard Model. A crucial requirement for precision physics is the possibility of defining statistically robust error bars, and controlling the systematic errors in the solution. Solutions need to be both precise and accurate, calling for rigorous uncertainty quantification.

More specifically, we consider here examples where we aim to determine a function defined on a one-dimensional real interval, which we denote $\mathcal {I}$, using a finite set of data points. In the absence of further hypotheses, these functions are elements of infinite-dimensional spaces, so that the inverse problem is necessarily ill-posed. In the absence of infinitely many data points, further assumptions are needed in order to get a solution.

We frame our presentation from a probabilistic point of view, where the quantities that need to be determined are promoted to stochastic variables and the solution to the inverse problem is provided in terms of probability distributions. The probabilistic approach has, in our opinion, two main advantages:

1. it spells out explicitly all the assumptions about the model in the prior probability distribution;
2. it yields a characterization of the possible solutions in terms of *posterior* probabilities.

The division between prior and posterior distributions is an appealing feature of the probabilistic approach, which clearly distinguishes the impact of any model assumption from the impact of the available data. We will see examples where we consider the impact of larger datasets, of more precise data, and of different choices of priors.

Building upon the mathematical literature, we address the solution of two inverse problems that are relevant for current particle phenomenology, namely

1. the determination of Parton Distribution Functions (PDFs) from experimental (or lattice) data;
2. the inversion of the Laplace transform that allows to extract spectral densities from Euclidean lattice correlators.

The lectures are organized as follows. The notation and the mathematical framework are introduced in Sect. 2. We then focus on three different methods to address the solution of the inverse problem that are widely in use in the particle phenomenology community. Section 3 is devoted to the Backus–Gilbert method; we discuss the basic idea behind the method and its applications, in particular for the extraction of spectral densities from lattice QCD correlator. Section 4 introduces the usage of Gaussian processes for inverse problems. Gaussian processes are a non-parametric method, which relies on Bayesian inference in order to deduce the posterior distribution for the solution of the inverse problem. Finally, Sects. 5, 6, and 7 are devoted to the solution of the inverse problem using Neural Networks. The solution based on Neural Networks is divided into three parts: in Sect. 5, we introduce the notation used and discuss the prior distribution of the output of the Neural Networks at initialization; Sect. 6 is devoted to the analysis of the training dynamics of Neural Networks, using the Neural Tangent Kernel; finally, in Sect. 7, we discuss the so-called lazy training regime, where an analytical solution can be obtained. Understanding in detail the training dynamics is a key ingredient in order to assess the robustness of the solution and to relate the solution obtained using Neural Networks to the ones obtained using other methods, like Backus–Gilbert or Gaussian Processes. In Sect. 8, we attempt a synthesis of the results obtained using the different methods, and we discuss the relations between them.

In writing these lectures, we had to make a number of choices about the topics to be covered and the level of detail. We have tried to provide a self-contained presentation, focusing on methods that are more familiar to us and that we have extensively used. In a limited amount of hours, it was impossible to provide an exhaustive overview of the field, and we apologize for the omissions.^1^ We hope nonetheless that these lectures will be useful for students and researchers interested in the solution of inverse problems in particle phenomenology, and more widely in computational science.

## Preliminaries

### Notation and definitions

Let us begin by introducing the notation used in these lectures.

***Input functions.***

The space of *input functions* is a Banach space, *X*. A Banach space is a *complete, normed* vector space. The actual space of functions that we want to explore depends on the problem at hand, but we always assume the existence of a metric that allows us to define the distance between two functions. The completeness of the space is important to prove mathematical theorems, but is not needed in practice for the problems considered here.

#### Example 1

The space of parton distribution functions (PDFs) is the space of functions

1 $$\begin{aligned} f{:}\, [0,1] \rightarrow \mathbb {R}, \end{aligned}$$

sometimes with the additional requirement that *f* is integrable, so that the sum rules can be satisfied. In *all* current determinations of the PDFs, see e.g. [2–4], a choice of functional form is made, which is defined by a set of $N_{\textrm{model}}$ parameters. Therefore, the set of input functions is effectively reduced to a finite-dimensional space spanned by these parameters. For example, one could use a basis of orthogonal polynomials or a neural network parametrization to represent the functions. These choices introduce a model dependence in the determination of the PDFs.

#### Example 2

The space of spectral densities is the space of functions

2 $$\begin{aligned} \rho {:}\, [0,\infty ) \rightarrow \mathbb {R}^+, \end{aligned}$$

with the additional requirement that ρ is integrable, and more specifically that

3 $$\begin{aligned} \int _0^\infty \textrm{d}\omega \, \rho (\omega ) = 1. \end{aligned}$$

Note that single particle stable states correspond to delta functions in the spectral density, which is therefore a distribution, not a function. We will come back to this point later.

#### Example 3

When considering the scattering of two particles, the amplitude can be decomposed in partial waves, so that the physical process is described by a set of functions $\mathcal {M}_{\ell }(\sqrt{s})$. It is customary to rewrite

4 $$\begin{aligned} \mathcal {M}_{\ell }^{-1} = \mathcal {K}_{\ell }^{-1} - i \rho , \end{aligned}$$

where ρ is the phase space factor and the K-matrix $\mathcal {K}_{\ell }$, which is a meromorphic function of $\sqrt{s}$. The space of input functions in this case is the space of meromorphic functions defined on the complex $\sqrt{s}$ plane.

***Forward map.***

The *forward map* maps the input functions to some output. The forward map is sometimes called the *model* and is denoted by $\mathcal {F}$

5 $$\begin{aligned} \mathcal {F}{:}\, X&\rightarrow R, \nonumber \\ f&\mapsto r = \mathcal {F}(f). \end{aligned}$$

Given a model $\mathcal {F}$ and an input function *f*, we can compute the output *r*. We assume that the space of output functions *R* is also a Banach space. The output functions are the *theoretical predictions* for the observables that are measured in the experiments.

#### Example 4

In the case of PDFs, the forward map that yields the theoretical prediction for the structure functions measured in a DIS experiment is

6 $$\begin{aligned} F(x,Q^2) = \mathcal {F}(f) = \int _0^1 \frac{\textrm{d}\xi }{\xi }\, C(x/\xi , Q^2)\, f(\xi ), \end{aligned}$$

where $C(x,Q^2)$ is the coefficient function that is known from perturbation theory. The coefficient functions can be computed at different orders in perturbation theory, yielding different (NLO, NNLO,...) theoretical predictions.

#### Example 5

The Laplace transform of the spectral densities yields the theoretical prediction for the Euclidean correlators

7 $$\begin{aligned} C(t) = \int _0^\infty \textrm{d}\omega \, e^{-\omega t}\, \rho (\omega ), \quad \text {for } t> 0. \end{aligned}$$

The lattice correlators are computed from Monte Carlo simulations of the Euclidean path integral. They are the data used to infer $\rho (\omega )$, just like the measurements of structure functions in the case of PDFs. The use of the letter *C* to denote the correlators is standard in the lattice community; they should not be confused with the coefficient functions $C(x,Q^2)$ in the case of PDFs discussed in the example above. Rather than changing a consolidated notation, we hope that the context will make it clear which of the two is being referred to.

#### Example 6

The scattering amplitudes determine the discrete levels of the energy spectrum of the theory in a finite volume. The forward map in this case is given by the Lüscher formula, which states that the energy levels are the solutions of the equation

8 $$\begin{aligned} \det \left[ \mathcal {K}^{-1}(E) + F(E,\textbf{P},L)\right] = 0, \end{aligned}$$

where $F(E,\textbf{P},L)$ is a known function of the energy, the total momentum, and the volume.

***Observables.***

The *observables* are the quantities that are measured in the experiments. The central values quoted by the experiments are denoted by *Y*. Experiments only ever measure a finite number of observables, $N_{\textrm{dat}}$, and hence

9 $$\begin{aligned} Y = \{ Y_I, \, I = 1, \ldots , N_{\textrm{dat}}\} \in \mathbb {R}^{N_{\textrm{dat}}}. \end{aligned}$$

Observables will be labeled by capital roman indices, I,J,…. At times, we will write all indices explicitly; however, in general, we suppress the indices and use a compact notation, where *Y* is a vector in $\mathbb {R}^{N_{\textrm{dat}}}$.

The corresponding theoretical predictions are denoted by *T* and are computed by applying the forward map at the points corresponding to the observables

10 $$\begin{aligned} T{:}\, X&\rightarrow \mathbb {R}^{N_{\textrm{dat}}},\nonumber \\ f&\mapsto T[f]. \end{aligned}$$

There is a theoretical prediction for each observable, i.e. $T_I[f] \in \mathbb {R}, I = 1, \ldots , N_{\textrm{dat}}$.

#### Example 7

In the case of PDFs, the observables are the structure functions measured at various kinematical points in a DIS experiment. The experimental data points are identified by the values of the Bjorken *x* and the virtuality $Q^2$. The observables are then defined as

11 $$\begin{aligned} Y_I = F(x_I, Q^2_I), \quad I = 1, \ldots , N_{\textrm{dat}}. \end{aligned}$$

The theoretical predictions are computed from the forward map as

12 $$\begin{aligned} T_I = T_I[f] = \int _0^1 \frac{\textrm{d}\xi }{\xi }\, C(x_I/\xi , Q_I^2)\, f(\xi ). \end{aligned}$$

In order to emphasize the linear dependence of the obervables on the PDFs, we can rewrite the equation above as

13 $$\begin{aligned} T_I = T_I[f] = \int _0^1 \textrm{d}\xi \, C_{I}(\xi )\, f(\xi ), \end{aligned}$$

where $C_{I}(\xi ) = C(x_I/\xi , Q_I^2)/\xi$ is the coefficient function evaluated at the kinematical point corresponding to the observable $Y_I$.

#### Example 8

When extracting spectral densities, we can only compute the Euclidean correlators for a finite number of time separations on a discretized spacetime lattice. Hence

14 $$\begin{aligned} Y_I = C(t_I), \quad I = 1, \ldots , N_{\textrm{dat}}, \end{aligned}$$

where in this case $N_{\textrm{dat}}$ is the number of discrete values of *t* where the measurement of the correlators yields a good signal—a more quantitative definition of *good* signal will be discussed below.

#### Example 9

In the case of scattering amplitudes, the observables are the discrete energy levels of the theory in a finite volume, which are measured in lattice simulations from the large-time behavior of Euclidean space correlators. The theoretical predictions for these energy levels are computed from the forward map as the solutions of the Lüscher formula in Eq. (8).

It is important to appreciate that in all these examples, we only have access to a finite number of observables, $N_{\textrm{dat}}$, and therefore, the information that can be extracted from the data is limited. When working with a finite number of measurements, often subject to noise, we cannot aim to fully reconstruct the input functions. Looking at the examples that we introduced above, we can anticipate that

1. in the case of PDFs, the measurements of the structure functions only cover a finite kinematic range, leading to large uncertainties in the so-called extrapolation regions;
2. in the case of spectral densities, measuring the correlators for a finite number of discrete values of the Euclidean time only allows the reconstruction of a limited number the states contributing to $\rho (\omega )$.
3. in the case of scattering amplitudes, the quantization condition only allows the computation of the amplitude at a finite number of discrete energy values. The form of the amplitude in between these values is not constrained by the data and the reconstruction is regulated by the choice of prior [5].

Experimental data are subject to *observational noise*, which is modeled as

15 $$\begin{aligned} Y_I = T_I[f] + \eta _I, \quad I = 1, \ldots , N_{\textrm{dat}}, \end{aligned}$$

where η is a random variable in $\mathbb {R}^{N_{\textrm{dat}}}$ with probability distribution $P_\eta$. Unless otherwise stated, we assume that $P_\eta$ is a multivariate Gaussian distribution, centered at the origin and with a covariance matrix $C_Y$, given by the experiments

16 $$\begin{aligned} P_\eta (\eta ) = \frac{1}{\sqrt{|2 \pi C_Y|}}\, e^{-\frac{1}{2} \eta ^T C_Y^{-1} \eta }. \end{aligned}$$

The central values quoted by the experiments are one particular draw from the probability distribution $P_\eta$.

### A solution on a grid

For the case of observables that depend linearly on the input function, a simple solution to the inverse problem is obtained by minimizing the $\chi ^2$, defined as

17 $$\begin{aligned} \chi ^2[f] = \sum _{I,J=1}^{N_{\textrm{dat}}} \left( Y_I - T_I[f]\right) \left( C_Y^{-1}\right) _{IJ} \left( Y_J - T_J[f]\right) , \end{aligned}$$

or equivalently suppressing the indices

18 $$\begin{aligned} \chi ^2[f] = (Y - T[f])^T C_Y^{-1} (Y - T[f]). \end{aligned}$$

The solution is then given by the function $\hat{f}$ that minimizes $\chi ^2[f]$

19 $$\begin{aligned} \hat{f} = \arg \min _f \chi ^2[f]. \end{aligned}$$

We present a solution to this problem, obtained by discretizing the input function on a grid of $N_{\textrm{grid}}$ points, and then minimizing $\chi ^2$ with respect to the values of the function at these points. The unknown function is, therefore, represented as a vector of $N_{\textrm{grid}}$ real numbers, $f \in \mathbb {R}^{N_{\textrm{grid}}}$, while the forward map is represented as a $N_{\textrm{dat}}\times N_{\textrm{grid}}$ matrix

20 $$\begin{aligned} T[f] = (\textrm{FK})f, \end{aligned}$$

where $(\textrm{FK})$ is the matrix representation of the forward map. Note that in this framework, we have reduced the infinite-dimensional space of input functions to a $N_{\textrm{grid}}$ dimensional real space.

The covariance matrix $C_Y$ is positive-definite, its SVD decomposition yields

21 $$\begin{aligned} C_Y = R\, \Sigma ^{1/2}\, \Sigma ^{1/2} R^T, \end{aligned}$$

where *R* is orthogonal and Σ is the diagonal matrix made of the eigenvalues of $C_Y$. We can then rewrite $\chi ^2$ as

22 $$\begin{aligned} \chi ^2[f]&= (Y - (\textrm{FK})f)^T R\, \Sigma ^{-1/2}\, \Sigma ^{-1/2} R^T (Y - (\textrm{FK})f) \nonumber \\&= \Vert \Upsilon - A f \Vert ^2, \end{aligned}$$

where we have introduced the uncorrelated, whitened data Υ and the rescaled forward map *A*

23 $$\begin{aligned} \Upsilon = \Sigma ^{-1/2} R^T Y, \quad A = \Sigma ^{-1/2} R^T (\textrm{FK}). \end{aligned}$$

The solution to the minimization problem in Eq. (19) is then given by acting with the pseudo-inverse of *A*, denoted as $A^+$, on the rescaled data [6]

24 $$\begin{aligned} \hat{f} = A^+ \Upsilon . \end{aligned}$$

Using the following identity:

25 $$\begin{aligned} A^+ = (A^T A)^{+} A^T, \end{aligned}$$

allows us to rewrite the solution as

26 $$\begin{aligned} \hat{f} = M^{+} (\textrm{FK})^TC_Y^{-1} Y, \end{aligned}$$

where we have introduced the matrix *M*

27 $$\begin{aligned} M = (\textrm{FK})^TC_Y^{-1} (\textrm{FK}). \end{aligned}$$

In the physical applications of interest in these lectures, we will encounter two situations

- the matrix *M* is not invertible, and then, the solution in Eq. (26) is not unique;
- or *M* has a large condition number, and the solution is unstable with respect to small variations of the data.

Note that a non-invertible matrix *M* has a non-trivial kernel

28 $$\begin{aligned} \textrm{Ker}(M) = \{ f \in \mathbb {R}^{N_{\textrm{grid}}}{:}\,M f = 0 \}, \end{aligned}$$

which coincides with the kernel of the forward map, $\textrm{Ker}((\textrm{FK}))$. In this case, the solution is not unique, since any function of the form

29 $$\begin{aligned} \hat{f} + f_{\parallel }, \quad f_{\parallel } \in \textrm{Ker}(M)\, \end{aligned}$$

is also a solution. In both examples, in order to get a well-defined, stable solution, some form of regularization is needed.

### Probabilistic framework

As we will discuss later in the lectures, it is useful to work in a probabilistic framework, where the unknown function *f* is promoted to a stochastic process and the answer of the inverse problem is given in the form of a posterior probability for the solution *f*. In general, this is a probability measure in an infinite-dimensional space of functions and needs to be handled with (mathematical) care. In practice, following the discussion in the above section, we aim to compute the joint posterior distributions^2^

30 $$\begin{aligned} \tilde{P}(f_1, \ldots , f_{N_{\textrm{grid}}}) = P(f_1, \ldots , f_{N_{\textrm{grid}}} | D_{N_{\textrm{dat}}}), \end{aligned}$$

for any generic set of values of *f*

31 $$\begin{aligned} f_\alpha = f(x_\alpha ), \quad \alpha = 1, \ldots , N_{\textrm{grid}}, \quad x_\alpha \in \mathcal {I}, \end{aligned}$$

and $\mathcal {I}$ is the domain of the function *f*. Looking at the two examples we discussed, we have

- $\mathcal {I} = [0,1]$ for the PDFs, since the Bjorken *x* is defined in this range;
- $\mathcal {I} = [0,\infty )$ for the spectral density, since the energy of the states contributing to $\rho (\omega )$ is positive.

We can marginalize with respect to some of the values of *f* and get the posterior distribution for a subset of the values of *f*

32 $$\begin{aligned} P_{\alpha _1 \ldots \alpha _n}(f_{\alpha _1}, \ldots , f_{\alpha _n} | D_{N_{\textrm{dat}}}) = \int \prod _{\alpha \ne \alpha _1, \ldots , \alpha _n} \textrm{d}f_\alpha \, P(f_1, \ldots , f_{N_{\textrm{grid}}} | D_{N_{\textrm{dat}}}). \end{aligned}$$

The central value and the variance of $f(x_\alpha )$ at a single point on the grid $x_{\alpha }$ are computed from the posterior $P_\alpha (f | D_{N_{\textrm{dat}}})$ as

33 $$\begin{aligned} \bar{f}_\alpha&= \mathbb {E}[f(x_\alpha )] = \int \textrm{d}f P_\alpha (f | D_{N_{\textrm{dat}}})\, f, \end{aligned}$$

34 $$\begin{aligned} \sigma _\alpha ^2&= \mathbb {E}[\left( f_\alpha - \bar{f}_\alpha \right) ^2] = \int \textrm{d}f P_\alpha (f | D_{N_{\textrm{dat}}})\, \left( f - \bar{f}_\alpha \right) ^2. \end{aligned}$$

Correlations between the values of the function *f* at multiple points can be computed using the joint distributions in Eq. 32.

## Backus–Gilbert method

### Backus–Gilbert method

We begin this section by reviewing the general principles behind the Backus–Gilbert method, which is a widely used approach to solve inverse problems in a non-parametric way. The method was originally developed in the context of geophysics [7], but has found applications in many other fields, including particle physics and lattice QCD. We follow here the presentation in Ref. [8], which leads naturally to a connection with Gaussian Processes, as we will see in the next section.

We start by considering the continuous version of the inverse problem, where we want to determine a function *f*(*x*) defined on a real interval $\mathcal {I}$, from a finite set of data points $D = \{Y_I, I=1, \ldots , N_{\textrm{dat}}\}$. The Backus–Gilbert method provides a way to construct an estimator $\hat{f}^{A}(x)$ for the function *f*(*x*), which is a linear combination of the data points

35 $$\begin{aligned} \hat{f}^{A}(x) = \sum _{I=1}^{N_{\textrm{dat}}} A_I(x) Y_I, \end{aligned}$$

defined by choosing a set of coefficients $A_I(x)$. For data that depend linearly on the function *f*(*x*), in the absence of statistical noise

36 $$\begin{aligned} Y_I = \int _{\mathcal {I}} \textrm{d}x' \, C_I(x') f(x'), \end{aligned}$$

where $C_I(x)$ are known functions that define the linear forward map for the problem under study.^3^ The Backus–Gilbert estimator can be rewritten as

37 $$\begin{aligned} \hat{f}^{A}(x) = \int _{\mathcal {I}} \textrm{d}x' \, \mathcal {R}^{A}(x,x') f(x'), \end{aligned}$$

where we introduced the *resolution function*

38 $$\begin{aligned} \mathcal {R}^{A}(x,x') = \sum _{I=1}^{N_{\textrm{dat}}} A_I(x) C_I(x'). \end{aligned}$$

Equation (37) shows explicitly that the BG estimator $\hat{f}^{A}(x)$ is a smeared version of the true solution *f*(*x*), where the smearing is dictated by the resolution function $\mathcal {R}^{A}(x,x')$.

In general, the coefficients $A_I(x)$ can be chosen by requiring that the resolution function $\mathcal {R}^{A}(x,x')$ is as *close* as possible to some target smearing function of our choice, which depends on the specific problem that is being solved. In order to quantify the closeness of $\mathcal {R}^{A}(x,x')$ to the target smearing function, we introduce a metric in the space of functions

39 $$\begin{aligned} d[u,v] = \Vert u-v \Vert _k, \end{aligned}$$

where the norm is defined with respect to a symmetric, positive-definite kernel $k(x,x^{\prime })$ as

40 $$\begin{aligned} \Vert u \Vert _k^2 = \int _{\mathcal {I}} \textrm{d}x\, \textrm{d}x'\, u(x) k(x,x') u(x'). \end{aligned}$$

Note that the arbitrariness in the choice of the kernel $k(x,x^{\prime })$ can be used to encode some prior information about the function *f*(*x*).

***Point estimation.***

In order to get a point estimator for the function *f*(*x*), we can choose the target smearing function to be as close as possible to a delta function $\delta (x-x^{\prime })$, which would correspond to an ideal estimator that recovers the true function *f*(*x*) without any smearing. In practice, this is done by minimizing the distance between the resolution function $$\mathcal {R}^{A}(x,x')$$ and the delta function $\delta (x-x^{\prime })$

41 $$\begin{aligned} d\left[ \mathcal {R}^{A}(x,x'), \delta (x-x')\right] ^2, \end{aligned}$$

as a function of the coefficients $A_I(x)$. Imposing

42 $$\begin{aligned} \frac{\partial }{\partial A_I(x)} d\left[ \mathcal {R}^{A}(x,x'), \delta (x-x')\right] ^2 = 0, \end{aligned}$$

yields

43 $$\begin{aligned} W_{IJ} A_J(x) = R_I(x), \end{aligned}$$

where

44 $$\begin{aligned} W_{IJ}&= \int _{\mathcal {I}} \textrm{d}x\, \textrm{d}x'\, C_I(x) k(x,x') C_J(x'), \end{aligned}$$

45 $$\begin{aligned} R_I(x)&= \int _{\mathcal {I}} \textrm{d}x'\, k(x,x') C_I(x'). \end{aligned}$$

Having introduced the matrix *W* and the vector *R* with explicit observable indices, we are going to suppress the indices and use a compact notation, where *W* is a matrix in the space of observables and *R* is a vector in the same space. Assuming that the matrix *W* is invertible, we can solve for the coefficients *A*(*x*) as

46 $$\begin{aligned} A(x) = R(x)^T W^{-1}, \end{aligned}$$

where we also suppressed the index for the coefficients $A_I(x)$, which is now implicit in the matrix–vector multiplication. Plugging this solution into Eq. (35) yields the point estimator for the function *f*(*x*)

47 $$\begin{aligned} \hat{f}^{\textrm{BG}}(x) = R(x)^T W^{-1} Y. \end{aligned}$$

The resolution function for this estimator is given by

48 $$\begin{aligned} \mathcal {R}^{\textrm{BG}}(x,x') = R(x)^T W^{-1} C(x'), \end{aligned}$$

where we introduced the vector *C*(*x*) with components $C_I(x)$.

***Noisy data.***

Statistical noise in the data is modeled by adding a stochastic term η to the theoretical prediction

49 $$\begin{aligned} Y_I = \int _{\mathcal {I}} \textrm{d}x'\ C_I(x') f(x') + \eta _I, \end{aligned}$$

where η is a vector of random variables $\eta _I$ with zero mean and covariance matrix $C_Y$. The point estimator is obtained by minimizing the regulated distance

50 $$\begin{aligned} d\left[ \mathcal {R}^{A}(x,x'), \delta (x-x')\right] ^2 + \lambda A^T(x) C_Y A(x), \end{aligned}$$

where λ is a regularization parameter that controls the trade-off between the closeness of the resolution function to the delta function and the stability of the solution with respect to noise in the data. The directions in data space that correspond to the larger eigenvalues of the covariance matrix $C_Y$ are more affected by noise, and the regularization term in Eq. (50) suppresses the contribution of these directions to the solution. The solution for the coefficients $A_I(x)$ is given by

51 $$\begin{aligned} A(x)^T = R(x)^T \left( W + \lambda C_Y\right) ^{-1}, \end{aligned}$$

and the point estimator is given by

52 $$\begin{aligned} \hat{f}^{\textrm{BG}}(x) = R^T(x) \left( W + \lambda C_Y\right) ^{-1} Y. \end{aligned}$$

The resolution function for this estimator is given by

53 $$\begin{aligned} \mathcal {R}^{\textrm{BG}}(x,x') = R^T(x) \left( W + \lambda C_Y\right) ^{-1} C(x'). \end{aligned}$$

Note that, because the covariance matrix $C_Y$ is positive-definite, the regularized matrix $W + \lambda C_Y$ is also positive-definite and therefore invertible, so that the solution in Eq. (51) is well defined for any value of the regularization parameter λ>0.

#### Exercise 1

Derive the solution for the case of noisy data, starting from the minimization of the regulated distance in Eq. (50). If needed, write all the indices explicitly to get familiar with the notation.

***A comment on the probabilistic interpretation.***

Note that the BG solution does not start from a prior distribution for the solution. However, we can construct a posterior distribution for the solution by bootstrapping the point estimator discussed above over the noise in the data. In this way, the statistical fluctuations of the data translate into fluctuations of the solution.

### BG on a grid

It is instructive to pause for a second and rewrite the BG derivation for the case discussed in Sect. 2.2, where the solution is represented as the finite-dimensional vector of values $f_\alpha$, and the theoretical prediction is computed as a matrix–vector multiplication

54 $$\begin{aligned} T[f] = (\textrm{FK})f. \end{aligned}$$

The estimator for the solution is a linear combination of the data

55 $$\begin{aligned} \hat{f}^A(x_\alpha ) = \hat{f}^A_\alpha = \sum _{I=1}^{N_{\textrm{dat}}} A_{\alpha I} Y_I, \end{aligned}$$

where the functions $A_I(x)$ that we encountered in the continuous case are now replaced by the coefficients $A_{\alpha I}$, i.e., by a matrix of coefficients *A* with dimensions $N_{\textrm{grid}}\times N_{\textrm{dat}}$. The norm in the space of functions is replaced by a norm in the space of vectors, defined in terms of a positive-definite matrix $K_{\alpha _1\alpha _2}$ as

56 $$\begin{aligned} \Vert u \Vert _K^2 = u^T K u. \end{aligned}$$

Similarly, we introduce the matrices *W* and *R* as

57 $$\begin{aligned} W_{IJ}&= (\textrm{FK})_{I\alpha _1} K_{\alpha _1\alpha _2} (\textrm{FK})^T_{\alpha _2 J}, \end{aligned}$$

58 $$\begin{aligned} R_{I\alpha }&= (\textrm{FK})_{I\alpha _1} K_{\alpha _1\alpha }. \end{aligned}$$

The interested reader can verify that the solution for the coefficients $A_{\alpha I}$ for the case of noisy data is given by

59 $$\begin{aligned} A = R^T \left( W + \lambda C_Y\right) ^{-1}. \end{aligned}$$

#### Exercise 2

Derive the solution for the case of noisy data, starting from the minimization of the regulated distance in Eq. (50), with the appropriate replacements for the discrete case.

The point estimator for the solution is given by

60 $$\begin{aligned} \hat{f}^{\textrm{BG}} = R^T \left( W + \lambda C_Y\right) ^{-1} Y, \end{aligned}$$

and the resolution function is given by

61 $$\begin{aligned} \mathcal {R}^{\textrm{BG}}&= R^T \left( W + \lambda C_Y\right) ^{-1} (\textrm{FK})\end{aligned}$$

62 $$\begin{aligned}&= K (\textrm{FK})^T\left( (\textrm{FK})K (\textrm{FK})^T+ \lambda C_Y\right) ^{-1} (\textrm{FK}). \end{aligned}$$

We have suppressed all the indices in the equations above. It may be useful to rewrite the resolution function with all the indices explicitly written as a way to get familiar with the notation. These expressions will be useful in connecting the BG result with other solutions that we will discuss in the next sections.

### Closure tests

Closure tests are a powerful tool to validate the solutions of the inverse problem, and to assess the quality of the reconstructed function. The idea is to generate synthetic data from a known solution, and then to apply, in this particular case, the BG method to these data to see if we can recover the original solution. This allows us to check that the implementation of the BG method is correct, and to understand how well the method performs in practice. The synthetic data are generated by applying the forward operator to a known solution, $f_0$, and adding noise to mimic the statistical uncertainty of the data. Using the grid notation introduced in Sect. 3.2, the synthetic data are generated as

63 $$\begin{aligned} Y = (\textrm{FK})f_0 + \epsilon , \end{aligned}$$

where $f_0$ is the known solution, $(\textrm{FK})$ is the forward operator, and ϵ is a vector of random variables with zero mean and covariance matrix $C_Y$. The BG method is then applied to the synthetic data *Y* to obtain an estimator $\hat{f}^{\textrm{BG}}$ for the solution and compare it with the known solution $f_0$.

Writing explicitly all the matrices that appear in the BG solution in Eq. (60) yields

64 $$\begin{aligned} \hat{f}^{\textrm{BG}}&= K (\textrm{FK})^T\left( (\textrm{FK})K (\textrm{FK})^T+ \lambda C_Y\right) ^{-1} (\textrm{FK})f_0 \nonumber \\&\qquad + K (\textrm{FK})^T\left( (\textrm{FK})K (\textrm{FK})^T+ \lambda C_Y\right) ^{-1} \epsilon . \end{aligned}$$

The first term on the right-hand side of the equation above is the contribution to the BG solution that comes from the known solution $f_0$, while the second term is the contribution that comes from the noise in the data. The second contribution is linear in the noise ϵ and therefore averages to zero over an ensemble of instances of the noise. The first contribution, as already discussed in Sect. 3.1, is a smeared version of the known solution $f_0$. A few algebraic manipulations allow us to rewrite the BG solution as

65 $$\begin{aligned} \hat{f}^{\textrm{BG}}&= \mathcal {R}^{\textrm{BG}} f_0 = K M \frac{1}{K M + \lambda } f_0, \end{aligned}$$

where

66 $$\begin{aligned} M = (\textrm{FK})^TC_Y^{-1} (\textrm{FK}), \end{aligned}$$

is the matrix we already introduced in Eq. (27). If *M* were invertible, then we would recover the exact solution $f_0$ in the limit $\lambda \rightarrow 0$. As discussed in Sect. 2, the matrix *M* is not invertible, and the BG solution is *one* prescription to regularize the problem. In the case where *M* is invertible but ill-conditioned, the BG solution for nonvanishing λ is a Tikhonov regularization of the problem. Equation (65) shows explicitly that the BG prescription yields a smeared version of the solution $f_0$, where the smearing is dictated by the matrix $K M \frac{1}{K M + \lambda }$.

### Lattice spectral densities and the HLT method

A contemporary application of the BG method is in the determination of spectral densities from Euclidean correlators computed in lattice field theories. Here, we focus on the HLT method as introduced in Ref. [9], which is a particular implementation of the BG method, tailored to the specific problem of spectral density reconstruction. We begin by formulating the problem and introducing the notation that is specific to this application. We follow the notation used in the original publication, which is different from the one we used in the general formulation of the problem in Sects. 2.1, 3.1, and 3.2.

***Observables.***

The observables used to extract the spectral densities are Euclidean correlators of field operators, which are defined as

67 $$\begin{aligned} C_L(t) = \frac{1}{L^3} \sum _{\vec {x}} \langle O(t,\vec {x}) O(0) \rangle _L, \end{aligned}$$

where *L* is the spatial extent of the lattice, and t>0 is the Euclidean time. The subscript *L* is a reminder of the fact that these correlators are computed in a finite volume. They are evaluated as the mean over an ensemble of gauge configurations generate by Monte Carlo sampling. Their covariance matrix is also computed from the available sample and scales like the inverse of the number of statistically independent configurations. The correlators are computed for a *finite* set of discrete time points t=a,2a,…,T, where *a* is the lattice spacing and *T* is the temporal extent of the lattice in physical units.^4^ The index *I* used to label the data points in the previous sections can be identified with the time index $t_I$ of the correlators, so that we can write $Y_I = C_L(t_I)$.

***The unknown function.***

The function that we want to determine as the solution of the inverse problem is the spectral density

68 $$\begin{aligned} \rho _L(E) = \frac{1}{L^3} \sum _{\vec {x}} \langle O(0,\vec {x})\, \delta (E - H_L)\, O(0)\rangle _L, \end{aligned}$$

where $H_L$ is the Hamiltonian of the theory in a finite volume. The spectral density is a positive-definite function of the energy *E*, and it contains information about the spectrum of the theory, as well as about the matrix elements of the operator *O* between the vacuum and the energy eigenstates. Comparing again with the notation used in the previous sections for the general formulation of the inverse problem, we can identify the unknown function *f*(*x*) with the spectral density $\rho _L(E)$. The argument of the unknown function in this case is the energy *E*; when we discretize the problem on a gird of points, the index α is used to label the grid points with the energy variable $E_\alpha$.

***Formulation of the problem.***

The Euclidean correlators, computed on a lattice with infinite time extent, and the spectral density are related by a Laplace transform

69 $$\begin{aligned} C_L(t) = \int _0^\infty \textrm{d}E\, e^{-tE} \rho _L(E). \end{aligned}$$

Note that in a finite volume, the spectrum of the Hamiltonian is discrete, which implies that the spectral density is a sum of delta functions

70 $$\begin{aligned} \rho _L(E) = \sum _{n} \rho _n(L) \delta (E - E_n), \end{aligned}$$

and therefore, it is a distribution rather than a regular function. As usual when dealing with distributions, we need to introduce a smearing function $\Delta _\sigma (E,E')$ in order to define a regular, smeared version of the spectral density

71 $$\begin{aligned} \hat{\rho }_L(\sigma ,E_*) = \int _0^\infty \textrm{d}E \, \Delta _\sigma (E,E_*) \rho _L(E). \end{aligned}$$

The smearing function $$\Delta _\sigma (E,E')$$ is a regular function, which is peaked around $E=E^{\prime }$ and has a width σ. The infinite-volume, unsmeared spectral density can be obtained by taking the limit $$\sigma \rightarrow 0$$ and $$L \rightarrow \infty$$ in the smeared spectral density

72 $$\begin{aligned} \rho (E) = \lim _{\sigma \rightarrow 0} \lim _{L \rightarrow \infty } \hat{\rho }_L(\sigma ,E), \end{aligned}$$

where the order of the limits matters.

Note that the limit $\sigma \rightarrow 0$ may not be needed in practice, since the smeared spectral density $\hat{\rho }_L(\sigma ,E)$ is a regular function of *E* that can be directly compared with experimental data, which can also be smeared.

***HLT formalism on a finite lattice.***

For correlators computed on a lattice with finite time extent *T*, the relation between the correlators and the spectral density is given by

73 $$\begin{aligned} Y_I = C_L(t_I) = \int _0^\infty \textrm{d}E\, b_T(t_I,E) \rho _L(E), \end{aligned}$$

where the basis function $b_T(t,E)$ is given by

74 $$\begin{aligned} b_T(t,E) = e^{-tE} + e^{-(T-t)E}. \end{aligned}$$

The basis function $b_T(t,E)$ is a generalization of the Laplace kernel $e^{-tE}$ that takes into account the finite time extent of the lattice. In the limit $T \rightarrow \infty$, the basis function $b_T(t,E)$ reduces to the Laplace kernel

75 $$\begin{aligned} \lim _{T \rightarrow \infty } b_T(t,E) = e^{-tE} . \end{aligned}$$

The function $b_T(t_I,E)$ is plays the role of the function $C_I(x)$ that we introduced in the general formulation of the inverse problem—remember that *I* identifies the data point and *E* is the argument of the unknown function.

***HLT solution.***

In the HLT formulation, as in all BG formulations, the solution is expanded as a linear combination of the data

76 $$\begin{aligned} \hat{\rho }_{L}(E^*) = \sum _{t=0}^{t_{\textrm{max}}} g_t(E^*)\, C(t+a), \end{aligned}$$

so that the inverse problem is reduced to determining the coefficients $g_t(E^*)$. As discussed in Sect. 3.1, any BG estimator yields a smeared version of the unknown function. In the notation of HLT, the smearing kernel is

77 $$\begin{aligned} \bar{\Delta }(E^*,E) = \sum _{t=0}^{t_{\textrm{max}}} g_t(E^*)\, b_T(t+a, E). \end{aligned}$$

The coefficients $g_t(E^*)$ are determined by requiring that the smearing kernel approximates a specific function, e.g.

78 $$\begin{aligned} \Delta _{\sigma }(E^*,E) = \frac{1}{\mathcal {N}} \, \exp \left[ - \frac{\left( E - E^*\right) ^2}{2\sigma ^2} \right] , \end{aligned}$$

with the *unusual* normalization

79 $$\begin{aligned} \mathcal {N} = \int _{0}^{\infty } \textrm{d}E\, \exp \left[ - \frac{\left( E - E^*\right) ^2}{2\sigma ^2} \right] . \end{aligned}$$

Following the derivation in Sect. 3.1, for each value of $E^*$, we minimize a cost function W[λ,g] with respect to the parameters $g_t(E^*)$. For each value of $E^*$, we thereby determine $(t_{\textrm{max}} + 1)$ coefficients, which are necessary to construct $\hat{\rho }_{L}(E^*)$ according to Eq. 76. which is the sum of the distance between $\Delta _{\sigma }$ and $\bar{\Delta }$ and a term that takes into account the covariance of the data

80 $$\begin{aligned} W\left[ \lambda , g\right] = \left( 1-\lambda \right) A[g] + \lambda \frac{B[g]}{C(0)^2}, \end{aligned}$$

where

81 $$\begin{aligned} A[g]&= \int _{E_0}^{\infty } \textrm{d}E\, \left| \Delta _{\sigma }(E^*,E) - \bar{\Delta }(E^*,E) \right| ^2\, \end{aligned}$$

82 $$\begin{aligned} B[g]&= \sum _{t,t'} g_t(E^*) \left( C_Y^{-1}\right) _{tt'} g_{t'}(E^*), \end{aligned}$$

and $C_Y$ is the covariance of the Euclidean correlators

83 $$\begin{aligned} \left( C_Y\right) _{tt'} = \textrm{Cov}[C(t), C(t')]. \end{aligned}$$

Note that the integral in Eq. (81) has a lower integration limit $E_0$, which cuts off the small values of *E*.

The two terms, *A*[*g*] and *B*[*g*], are the equivalent of the two terms that appear in Eq. 50; in the case of HLT, the coefficients are chosen to approximate a well-behaved smearing kernel rather than the singular delta distribution of Eq. (50). The main idea is that the HLT solution aims directly for a smeared solution, with the emphasis being on approximating the smearing kernel as best as possible, rather than trying to approximate a Dirac delta.

#### Exercise 3

Show that the solution of the minimization yields

84 $$\begin{aligned} g^{\textrm{HLT}}_t(E^*; \lambda , \sigma )&= W^{-1}(\lambda )_{tt'} \left[ f_{t'}(E^*; \lambda , \sigma ) + R_{t'} \frac{1 - R^T W^{-1}(\lambda ) f(E^*; \lambda ,\sigma )}{R^T W^{-1}(\lambda ) R} \right] , \end{aligned}$$

where we introduced

85 $$\begin{aligned}&R_t = \int _0^\infty \textrm{d}E\, b_T(t+a, E), \end{aligned}$$

86 $$\begin{aligned}&f_t(E^*; \lambda , \sigma ) = \left( 1 - \lambda \right) \, \int _{E_0}^\infty \textrm{d}E\, b_t(t+a, E) \Delta _{\sigma }(E^*, E), \end{aligned}$$

and

87 $$\begin{aligned} W(\lambda )_{tt'} = (1-\lambda ) M_{tt'} + \lambda \frac{(C_Y)_{tt'}}{C(0)^2}, \end{aligned}$$

where

88 $$\begin{aligned} M_{tt'} = \int _{E_0}^\infty \textrm{d}E\, b_T(t+a, E)\, b_T(t'+a, E). \end{aligned}$$

The matrix *M* is an ill-conditioned matrix; as discussed in Sect. 3.1 adding to the distance the *B* term, which takes into account the covariance of the data, acts as a regulator.

***A comment on*** *A*.

For $T\rightarrow \infty$, the function $b_T$ becomes an exponential, and therefore, the *A* term in the distance is

89 $$\begin{aligned} A[g] = \int _{E_0}^\infty \textrm{d}E\, \left| \sum _{t=0}^{t_{\textrm{max}}} g_t(E^*;\lambda , \sigma ) e^{-(t+a) E} - \Delta _{\sigma }(E^*,E) \right| ^2. \end{aligned}$$

***Some insight into the HLT solution.***

The HLT approach focuses on approximating the smearing kernel $\Delta _{\sigma }(E^*,E)$ as best as possible, rather than trying to find the best point estimator for the spectral density. It is possible, and desirable, to monitor the quality of the approximation of the smearing kernel using as an estimator

90 $$\begin{aligned} \delta _{\sigma }(E^*, E) = 1 - \frac{\bar{\Delta }(E^*,E)}{\Delta _\sigma (E^*,E)}. \end{aligned}$$

The quality of the approximation of the smearing kernel can be improved by increasing the number of data points, i.e., by increasing $t_{\textrm{max}}$. It is worthwhile to emphasize the quantitative character of this estimate; for a given quality of the kernel approximation, e.g., fixing

91 $$\begin{aligned} \delta _{\sigma }(E^*, E^*) = 0.05, \end{aligned}$$

allows to explore regions in the σ vs. $t_{\textrm{max}}$ plane where the approximation of the smearing kernel is good enough, and therefore to determine the optimal choice of σ for a given number of data points.

However, the quality of the approximation is also affected by the choice of the regularization parameter λ. Increasing λ improves the stability of the solution with respect to noise in the data, but it also worsens the approximation of the smearing kernel. The optimal choice of λ is, therefore, a trade-off between the quality of the approximation of the smearing kernel and the stability of the solution with respect to noise in the data.

## Gaussian processes

### A brief introduction to Gaussian processes

A *stochastic process* is a collection of random variables $$\{f(x): x \in \mathcal {I}\}$$, where $$\mathcal {I}$$ is the set on which *f* is defined. For any discrete set of points $$\{x_1, \ldots , x_n\}$$, the random variables $$\{f(x_1), \ldots , f(x_n)\}$$ have a joint probability distribution. A *Gaussian process* is a stochastic process, such that any finite collection of random variables has a multivariate Gaussian distribution. As such, a Gaussian process is completely specified by its mean function *m*(*x*) and its covariance function $k(x,x^{\prime })$, defined as

92 $$\begin{aligned} m(x) = \mathbb {E}[f(x)], \qquad k(x,x') = \mathbb {E}[(f(x) - m(x))(f(x') - m(x'))]. \end{aligned}$$

In the context of the inverse problems discussed in these lectures, we use Gaussian processes to define a prior distribution over functions and then use the observed data to obtain a posterior distribution, as suggested in Sect. 2.3.

***Setup of the problem.***

Following the conventions in Sect. 2.2, we represent the function *f* as an $N_{\textrm{grid}}$-dimensional real vector, made of the values of the function *f* at selected points on a grid:

93 $$\begin{aligned} f_{\alpha } = f(x_{\alpha })\, \quad \alpha = 1, \ldots , N_{\textrm{grid}}. \end{aligned}$$

These values are promoted to stochastic variables with a joint prior probability distribution that is a multi-dimensional Gaussian with respective mean and covariance given by

94 $$\begin{aligned} m_{\alpha }&= m(x_{\alpha }) = \mathbb {E}_{P}[f_{\alpha }], \end{aligned}$$

95 $$\begin{aligned} K_{\alpha _1\alpha _2}&= k(x_{\alpha _1},x_{\alpha _2}) = \textrm{Cov}_{P}[f_{\alpha _1},f_{\alpha _2}], \end{aligned}$$

where we introduced the subscript *P* to remind the reader that here we consider averages over the prior distribution *P*

96 $$\begin{aligned} P(f | h) = \frac{1}{\sqrt{\det \left( 2\pi K\right) }} \exp \left\{ - \frac{1}{2} \left( f-m\right) ^T K^{-1} \left( f - m\right) \right\} . \end{aligned}$$

The covariance matrix of the Gaussian prior in this section is denoted by *K*, a symbol we used in Sect. 3 to denote the metric in the space of functions. The reason for this choice is that the covariance of the prior distribution plays a similar role to the metric in the Backus–Gilbert method.

Any parameter that appears in the definitions of *m* and *k* is called an *hyperparameter* and is collectively denoted by *h*. For the PDF determinations that we are going to analyze later, we choose m(x)=0 and a Gibbs kernel

97 $$\begin{aligned} k(x,x') = \sigma ^2 \sqrt{\frac{2 \ell (x) \ell (x')}{\ell (x)^2 + \ell (x')^2}}\, \exp \left[ - \frac{(x-x')^2}{\ell (x)^2 + \ell (x')^2} \right] , \end{aligned}$$

where

98 $$\begin{aligned} \ell (x) = \ell _0 (x + \delta ). \end{aligned}$$

There are two hyperparameters in this kernel, σ and $$\ell _0$$, while δ is a small regulator, which is introduced in order to avoid the singularity at x=0. By inspecting Eq. (97), we see that $$\sigma ^2$$ is the variance of *f*(*x*), which is the same for all values of *x*. This is a *choice* that is encoded in the prior. Other choices are possible, including the possibility of an *x*-dependent prefactor. The correlation between two values *f*(*x*) and $f(x^{\prime })$ decreases exponentially with the square of the distance between *x* and $x^{\prime }$. The rate of the exponential decay is dictated by ℓ(x) and $\ell (x^{\prime })$. For this particular choice of the prior, ℓ(x) grows linearly with *x*, which implies that the correlation between points decreases as x→0. The values of *f* for nearby points at small *x* are almost uncorrelated, which implies that the function is free to fluctuate more than at higher values of *x*. We will discuss later how to constrain the hyperparameters σ and $$\ell _0$$.

Remembering that the theoretical predictions are computed by multiplying the vector *f* by the forward map $(\textrm{FK})$

99 $$\begin{aligned} T = (\textrm{FK})f, \end{aligned}$$

it is clear that these are also Gaussian variables, and one can readily show that the average and the covariance of the theoretical prediction *T*, induced by the prior probability distribution of *f*, are given by

100 $$\begin{aligned} E[T_I]&= (\textrm{FK})_{I\alpha } m_\alpha , \end{aligned}$$

101 $$\begin{aligned} \textrm{Cov}[T_I,T_J]&= (\textrm{FK})_{I\alpha _1} K_{\alpha _1\alpha _2} (\textrm{FK})^T_{\alpha _1J}. \end{aligned}$$

Clearly, *T* is a vector of dimensions $N_{\textrm{dat}}$, with components $T_I$, $I = 1, \ldots , N_{\textrm{dat}}$—there is one theoretical prediction for every data point.

Finally, we have $N_{\textrm{dat}}$ data points. Their central values, as given by the experiments, are denoted $Y_I$, while their fluctuations are assumed to be Gaussian, with a covariance matrix $C_Y$, which also taken from experimental analyses. The fluctuations of the data around the central values are described by a stochastic variable

102 $$\begin{aligned} \epsilon \sim \mathcal {N}\left( 0, C_Y\right) . \end{aligned}$$

Remember once again that ϵ is an $N_{\textrm{dat}}$-dimensional vector, with components $\epsilon _I$. The set of central values and the covariance matrix of the data together constitute what we call the *dataset* $D_{N_{\textrm{dat}}}$.

### The Gaussian process solution

The solution of the inverse problem in this formalism is encoded in the *joint* posterior distribution

103 $$\begin{aligned} \tilde{P}(f, h) = P\left( f, h | D_{N_{\textrm{dat}}}\right) . \end{aligned}$$

Using simple probabilities, we can factorize

104 $$\begin{aligned} P\left( f, h | D_{N_{\textrm{dat}}}\right) = P(f | h, D_{N_{\textrm{dat}}})\, P(h | D_{N_{\textrm{dat}}}); \end{aligned}$$

the joint posterior is the product of two factors. The first one is the posterior probability induced by the data at fixed values of the hyperparameters *h*, while the second factor is the probability of the hyperparameters given the data. Let us analyze each factor on the right-hand side in turn.

The **first factor** is computed using Bayesian inference

105 $$\begin{aligned} P(f | h, D_{N_{\textrm{dat}}}) = \frac{P(D_{N_{\textrm{dat}}} | f, h)\, P(f | h)}{P(D_{N_{\textrm{dat}}} | h)}. \end{aligned}$$

The denominator in Eq. (105) is just a normalization factor, independent of *f*, which can be computed a posteriori. We focus here on on the expression in the numerator, which is the product of two factors, the likelihood and the prior. The likelihood is the probability of the data given the function *f* and the hyperparameters *h*. Assuming that the data are normally distributed around the theoretical predictions, we have

106 $$\begin{aligned} P(D_{N_{\textrm{dat}}} | f, h) = \frac{1}{\sqrt{\det \left( 2\pi C_Y\right) }} \exp \left\{ - \frac{1}{2} \left( Y - (\textrm{FK})f\right) ^T C_Y^{-1} \left( Y - (\textrm{FK})f\right) \right\} . \end{aligned}$$

For linear data, i.e. for theoretical predictions that depend linearly on the function *f*, the likelihood is a Gaussian distribution in *f*. The prior is also a Gaussian distribution in *f*, as given by Eq. (96). The product of two Gaussian distributions is another Gaussian distribution, so that the posterior distribution at fixed hyperparameters is also a Gaussian distribution in *f*. The mean and the covariance of this posterior distribution can be computed analytically, and are given by

107 $$\begin{aligned} \tilde{m}&= m + K (\textrm{FK})^T\left( C_{YT}\right) ^{-1} (Y - (\textrm{FK})m), \end{aligned}$$

108 $$\begin{aligned} \tilde{K}&= K - K (\textrm{FK})^T\left( C_{YT}\right) ^{-1} (\textrm{FK})K, \end{aligned}$$

where we introduced the matrix $C_{YT}$

109 $$\begin{aligned} C_{YT} = C_Y + (\textrm{FK})K (\textrm{FK})^T. \end{aligned}$$

Note that, because $C_{YT}$ is positive-definite, the diagonal elements of $\tilde{K}$ are smaller than the diagonal elements of *K*. Recalling that the diagonal elements of *K* and $\tilde{K}$ are, respectively, the variances of the prior and posterior distributions, we see that the that Bayesian inference reduced the uncertainty on the function *f* at fixed hyperparameters. The information contained in the data leads to a reduction of the uncertainty on the function *f*.

It is useful to prove that

110 $$\begin{aligned} (\textrm{FK})^T\left[ C_Y + (\textrm{FK})K (\textrm{FK})^T\right] ^{-1} = \frac{1}{1 + M K} (\textrm{FK})^TC_Y^{-1}, \end{aligned}$$

with the usual definition of the matrix *M* in Eq. (27), and therefore note that the covariance of the posterior can be rewritten in a form that is more similar to the expression for the covariance of the BG solution

111 $$\begin{aligned} \tilde{K}^{-1} = K^{-1} + M. \end{aligned}$$

This expression makes it clear that the posterior covariance is the result of the combination of the prior covariance and the information contained in the data, as encoded in *M*. The posterior covariance is smaller than both the prior covariance and the covariance of the BG solution, which is given by $M^{-1}$, since $\tilde{K}^{-1}> K^{-1}$ and $\tilde{K}^{-1}> M$.

#### Exercise 4

Prove Eqs. (107), (108), (110), and (111).

Using Eq. (110), the mean of the posterior distribution can be rewritten as

112 $$\begin{aligned} \tilde{m} = m + K \frac{1}{1 + M K} (\textrm{FK})^TC_Y^{-1} (Y - (\textrm{FK})m). \end{aligned}$$

This expression makes it clear that the posterior mean is the result of the combination of the prior mean and the information contained in the data, with a corrections that is proportional to the difference between the data and the theoretical predictions computed using the prior mean. For m=0, we can rewrite the posterior mean as

113 $$\begin{aligned} \tilde{m} = \left[ K \frac{1}{1 + M K}\right] \, (\textrm{FK})^TC_Y^{-1} Y. \end{aligned}$$

***A note on Tikhonov regularization.***

By choosing a prior covariance of the form $K = \sigma ^2 I$, where λ is a positive parameter, we can rewrite the posterior mean as

114 $$\begin{aligned} \tilde{m} = \frac{1}{M + \lambda }\, (\textrm{FK})^TC_Y^{-1} Y, \end{aligned}$$

which is the Tikhonov regularized solution of the inverse problem, with regularization parameter $\lambda = 1/\sigma ^2$. The Tikhonov regularized solution is a particular case of the GP solution, characterized by a diagonal prior covariance, i.e. a prior that does not encode any correlation between the values of the function at different points.

The **second factor** in Eq. (104) is the probability of the hyperparameters given the data. This is computed using Bayes’ theorem as well

115 $$\begin{aligned} P(h | D_{N_{\textrm{dat}}}) = \frac{P(D_{N_{\textrm{dat}}} | h)\, P(h)}{P(D_{N_{\textrm{dat}}})}. \end{aligned}$$

Once again, we only need to focus on the numerator, since the denominator is just a normalization factor. At fixed values of the hyperparameters, the data can be modeled as

116 $$\begin{aligned} Y = (\textrm{FK})f + \epsilon , \end{aligned}$$

where *f* is a stochastic variable with distribution given by the prior, and ϵ is a stochastic variable with distribution given by Eq. (102). Therefore, the data are the sum of two Gaussian variables, and are itself a Gaussian variable

117 $$\begin{aligned} P(D_{N_{\textrm{dat}}} | h) = \frac{1}{\sqrt{\det \left( 2\pi C_{YT}\right) }} \exp \left\{ - \frac{1}{2} \left( Y - (\textrm{FK})m\right) ^T C_{YT}^{-1} \left( Y - (\textrm{FK})m\right) \right\} . \end{aligned}$$

Choosing a uniform prior for the hyperparameters, the posterior distribution of the hyperparameters is proportional to the likelihood in Eq. (117). The most likely values of the hyperparameters are those that maximize this likelihood. Note that $P(D_{N_{\textrm{dat}}} | h)$ is a complicated function of the hyperparameters, since $C_{YT}$ depends on *h* in a non-trivial way. However, the distribution can be sampled using standard Markov Chain Monte Carlo methods, which allow us to obtain a characterization of the posterior distribution of the hyperparameters. If the posterior distribution of the hyperparameters is sharply peaked around its maximum, we can approximate it with a delta function, and use the most likely values of the hyperparameters in Eqs. (107) and (108) to obtain an approximation of the posterior distribution of *f*.

### GP determination of one PDF

A simple application of the GP methodology is the determination of the PDF

118 $$\begin{aligned} T_3(x) = u(x) + \bar{u}(x) - d(x) - \bar{d}(x), \end{aligned}$$

using DIS data from the BCDMS experiment. The difference between the proton and the deuteron structure functions, $F_2^p - F_2^d$, is proportional to $T_3$ only, so that we can write

119 $$\begin{aligned} F_2^p(x,Q^2) - F_2^d(x,Q2) = C_{T_3} \otimes T_3(x). \end{aligned}$$

This is a simple example of an inverse problem in the realm of PDFs determinations, where only one PDF is involved, and the forward map is given by a convolution with a kernel that is known in perturbation theory. The solution of this problem can be obtained using the GP methodology described in the previous section.

All results presented here are obtained using synthetic data generated from a known solution. The approach follows very closely the results presented in Ref. [10]. After applying standard kinematic cuts, we have 248 data points for the structure functions with their corresponding covariance matrix, $C_Y$, as described by the experimental analyses [11]. In the case of $T_3$, we want to implement the fact that the function has to be integrable at x=0. This can be achieved by introducing and additional hyperparameter α, used to rescale the kernel as

120 $$\begin{aligned} k(x,x') \rightarrow \phi (x) \, k(x,x') \, \phi (x'), \quad \phi (x) = x^\alpha , \end{aligned}$$

and imposing $$\alpha \in (-1,0]$$.

**Fig. 1:**
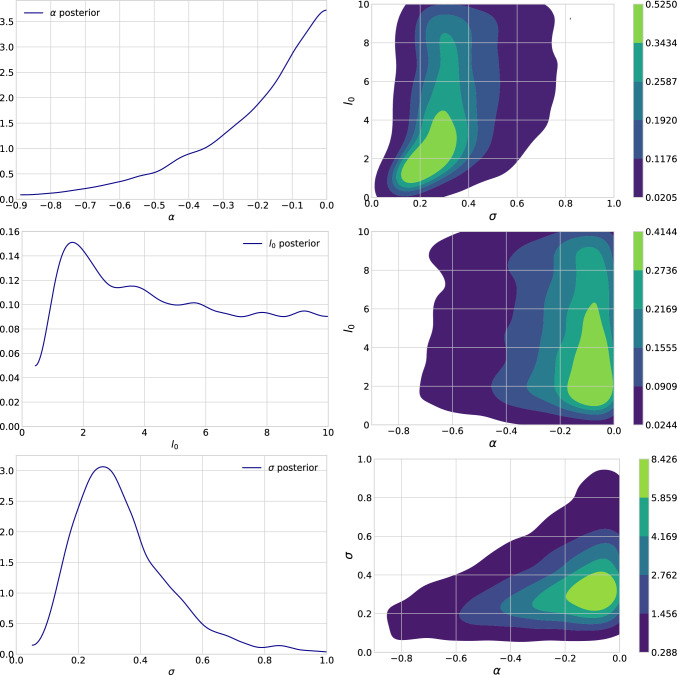
1-dimensional (left panel) and 2-dimensional (right panel) posteriors of the hyperparameters α, $l_0$ and σ. The hyperparameter σ is characterized by a sharply peaked posterior located around $\sigma \sim 0.25$ which quickly decays to zero (for this reason in the posterior plot only the region $\left( 0,1\right)$ is shown, even if the support of the prior is $\left( 0,10\right)$); α tends to sit closer to 0, with a slow decay for smaller values toward -1; the $l_0$ posterior discards the smaller values of the correlation length, it shows a peak for $l_0\sim 1.7$ and then remains fairly constant. (Plot taken from Ref. [10])

The Bayesian inference is performed in two steps. First, we sample the posterior of the hyperparameters, $P(h | D_{N_{\textrm{dat}}})$, using Markov Chain Monte Carlo methods. Then, for each set of hyperparameters drawn from this posterior, we use the analytical expressions in Eqs. (107) and (108) and generate a replica of $T_3$. The ensemble of replicas generated in this way provides a characterization of the posterior distribution of $T_3$, which can be used to compute the mean and the uncertainty of $T_3$ at any value of *x*. Results for the posterior distribution of the hyperparameters are shown in Fig. 1.

**Fig. 2:**
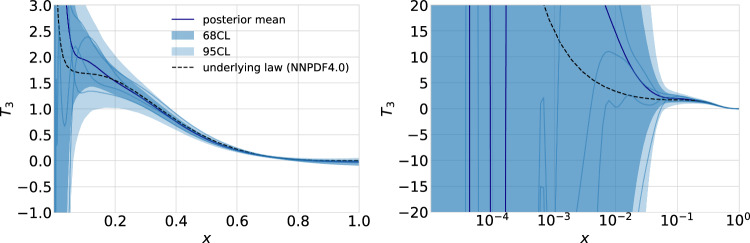
Samples from the posterior distribution of $T_3$, plotted in linear (left panel) and log (right panel) scale. The dark blue line represent the mean of the distribution, while the black dotted line the input PDF, $f_0$, used to generate pseudo-data. The shaded regions represent the 68CL and 95CL intervals, and in light blue, we plot a few representative samples from the distribution. The posterior displays a smaller variance in the regions sensitive to experimental data, and an increasing spread in the small and large-*x* extrapolation regions, where the results are mostly determined by the chosen prior. (Plot taken from Ref. [10])

The mean value and the uncertainty of $T_3$ obtained from the posterior distribution are shown in Fig. 2. The mean value is taken by averaging over the ensemble of replicas at each value of *x*, while the uncertainties are obtained from the 68% and 95% confidence intervals of the distribution of replicas. The uncertainty due to the fluctuations of the hyperparameters is included in the final uncertainty of $T_3$, since the replicas are generated by sampling the posterior distribution of the hyperparameters and then using the normal distribution of $T_3$ at fixed hyperparameters. The Bayesian inference is able to reconstruct the functional form of the PDF, as can be seen by comparing the dark blue and the dotted lines in the figure, which represent the mean of the posterior distribution and the input PDF, respectively. The uncertainty on $T_3$ is smaller in the regions where the data are more constraining, and increases in the extrapolation regions at small and large *x*, where the results are mostly determined by the choice of the prior.

### Closure tests

Following the discussion in Sect. 3.3, we can perform closure tests to validate the implementation of the GP method, and to assess the quality of the solution. Ignoring statistical fluctuations, i.e. setting $C_Y=0$, synthetic data are generated from a known solution, $f_0$, by applying the forward operator

121 $$\begin{aligned} Y = (\textrm{FK})f_0. \end{aligned}$$

In this case, the posterior mean is given by

122 $$\begin{aligned} \tilde{m} = K (\textrm{FK})^T\left[ (\textrm{FK})K (\textrm{FK})^T\right] ^{+} (\textrm{FK})\, f_0, \end{aligned}$$

where the superscript + denotes the pseudo-inverse, which is needed, since $(\textrm{FK})K (\textrm{FK})^T$ is not invertible when $C_Y=0$. By comparing with Eq. (64), we see that this solution corresponds to the solution obtained using the BG method with the kernel *K* as the metric. The two solutions coincide in the limit of zero noise when the hyperparameters are fixed to the values used to generate the data. It is interesting to note that, even when there is no statistical noise, the solution of the inverse problem does not coincide with the input function $f_0$, but it is a smeared version of it, with the same smearing kernel given by the resolution function of the BG method. The solution of the inverse problem is not exact, even in the absence of noise, because of the smoothing effect of the forward map and the regularization effect of the prior. The resolution function in this case is given by

123 $$\begin{aligned} \mathcal {R}^{\textrm{GP}}(x,x') = K(x,x') (\textrm{FK})^T\left[ (\textrm{FK})K (\textrm{FK})^T\right] ^{+} (\textrm{FK}). \end{aligned}$$

A nonvanishing experimental error can be readily added to the closure test by adding a noise term to the data, which is normally distributed around zero, with a covariance given by $C_Y$. In this case, the mean of the posterior distribution is given by Eq. (107) and the reconstruction kernel is

124 $$\begin{aligned} \mathcal {R} = K\, (\textrm{FK})^T\left[ (\textrm{FK})K (\textrm{FK})^T+ C_Y\right] ^{-1} (\textrm{FK}), \end{aligned}$$

so that the posterior mean and covariance can be rewritten as

125 $$\begin{aligned}&\tilde{m} - f_0 = \left[ \mathcal {R} - 1\!\!1\right] f_0 + a^T \epsilon , \end{aligned}$$

126 $$\begin{aligned}&\tilde{K} = \left( 1\!\!1 - \mathcal {R}\right) K \left( 1\!\!1 - \mathcal {R}\right) ^T + a^T C_Y a, \end{aligned}$$

where we have introduced

127 $$\begin{aligned} a^T = K\,(\textrm{FK})^T\left[ (\textrm{FK})\, K \, (\textrm{FK})^T+ C_Y\right] ^{-1}, \end{aligned}$$

so that

128 $$\begin{aligned} \mathcal {R} = a^T (\textrm{FK}). \end{aligned}$$

**Fig. 3:**
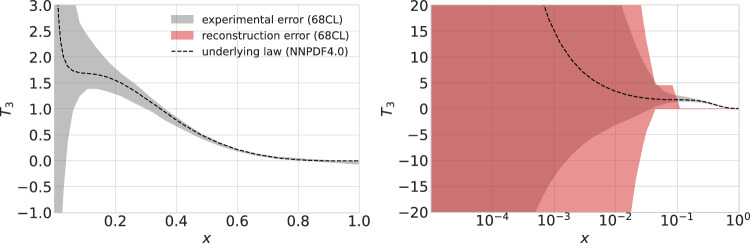
GP reconstruction of the input function $f_0$, with the 68% CL band for the experimental and reconstruction errors plotted separately in gray and red, respectively, according to Eq. (126). The error bands are overlapped rather than stacked. Plot taken from Ref. [10]

The decomposition in Eqs. (125), (126) highlights the fact that there are two types of contributions to the bias and to the posterior covariance matrix. The first term in both Eqs. (125) and (126) comes from the limited reconstruction of the central value and indeed would vanish when $\mathcal {R}=1\!\!1$. If $\mathcal {R} \ne 1\!\!1$, this term survives in the limit where $C_Y \rightarrow 0$, i.e. in the limit of no experimental errors on the data. The second term is the propagation of the covariance of the data into the covariance of the model. In the case $\mathcal {R} = 1\!\!1$, the only error fluctuations in the posterior distribution come from this term. In Fig. 3, Monte Carlo samples generated according to the reconstruction and experimental components are plotted separately for the BCDMS results, in red and gray, respectively. In the medium-*x* region, where more experimental data are available, the PDF uncertainty is dominated by the experimental error, yet a smaller reconstruction error is still present; when moving to the small and large-*x* extrapolation regions, the reconstruction error becomes the dominant one, pointing out the lack of experimental information. We stress once more how these qualitative considerations are precisely quantified in Eqs. (125), (126), giving the analytical expression for the posterior covariance matrices associated with the experimental and reconstruction error, making it possible to quote different component of the PDF error in the context of a phenomenology analysis.

Note that in this formalism, it is straightforward to assess the impact of a reduced uncertainty on the data, simply by reducing the covariance matrix $C_Y$ and recomputing the posterior distribution.

### GP for spectral densities

Let us now discuss how to apply the GP formalism to the problem of reconstructing spectral densities from Euclidean correlators computed on the lattice. The notation in this section follows very closely the one used in Ref. [12].

#### GP solution and connection with BG

Following the formalism introduced in this section, we represent the spectral density as a stochastic field $\rho (\omega )$, which is described by a GP centered around $\rho ^{\textrm{prior}}(\omega )$ and with covariance $\mathcal {K}^{\textrm{prior}}(\omega ,E)$. The prior probability measure is therefore

129 $$\begin{aligned} \Pi [\rho ] = \frac{1}{\mathcal {N}} \, \exp \biggr ( -\frac{1}{2} \left| \rho - \rho ^{\textrm{prior}} \right| ^2_{\mathcal {K}^{\textrm{prior}}} \biggr ) \;, \end{aligned}$$

where

130 $$\begin{aligned} \left| \rho - \right. \left. \rho ^{\textrm{prior}} \right| ^2_{\mathcal {K}^{\textrm{prior}}} = \int \textrm{d}E_1 \int \textrm{d}E_2 \left[ \rho (E_1) - \rho ^{\textrm{prior}}(E_1) \right] \mathcal {K}_\textrm{prior}^{-1}(E_1,E_2) \left[ \rho (E_2) - \rho ^{\textrm{prior}}(E_2) \right] , \end{aligned}$$

and the normalization

131 $$\begin{aligned} \mathcal {N} = \int \mathcal {D} \rho \; \Pi [\rho ]. \end{aligned}$$

In the previous expressions, $\mathcal {D} \rho$ represents the functional integration measure over the field variable ρ. By definition, the expectation value for ρ(E) from the prior distribution is

132 $$\begin{aligned} \rho ^{\textrm{prior}}(\omega ) = \int \mathcal {D}\rho \, \Pi [\rho ] \; \rho (\omega ). \end{aligned}$$

We then introduce the noise $\vec {\eta } \in \mathbb {R}^{\tau _\textrm{max}}$ which takes into account the uncertainty in the lattice data and is represented as a real-valued stochastic variable, for which we assume a multivariate Gaussian distribution with zero mean

133 $$\begin{aligned} \mathbb {G}[\vec {\eta }; C_Y] = \frac{1}{\sqrt{\text {det} (2\pi C_Y)}} \exp \left( -\frac{1}{2} \vec {\eta }\; C_Y^{-1} \; \vec {\eta } \right) , \end{aligned}$$

where $C_Y$ is the covariance matrix of the correlators. We also introduce a stochastic variable associated with the lattice data, $\vec {{\mathcal {C}}} \in \mathbb {R}^{\tau _{\textrm{max}}}$ with entries

134 $$\begin{aligned} \mathcal {C}(t) = \int \textrm{d}E \, b_T(t,E) \, \rho (E) + \eta (t). \end{aligned}$$

Given the distributions of Eqs. (129) and (134), we can evaluate the covariance associated to the variable $\vec {\mathcal {C}}$. It is straightforward to show that

135 $$\begin{aligned} \begin{aligned} \left\langle \mathcal {C}(t_1)\mathcal {C}(t_2) \right\rangle&= \int {\rm d}\vec {\eta }\;\mathcal {D}\rho \; \mathcal {C}(t_1) \, \mathcal {C}(t_2) \; \mathbb {G}[\vec {\eta }, \text {Cov}_d] \, \Pi [\rho ] \, \\&= \Sigma _{t_1t_2} + \left( C_Y\right) _{t_1t_2} \;, \end{aligned} \end{aligned}$$

where we defined

136 $$\begin{aligned} \Sigma _{t_1t_2} = \int \textrm{d}E_1 \int \; \textrm{d}E_2 \; b_T(t_1,E_1) \, \mathcal {K}^{\textrm{prior}}(E_1,E_2) \, b_T(t_2,E_2). \end{aligned}$$

In order to predict the value of the spectral density at the energy ω, we need to extend the dimensionality of the covariance of Eq. (135) to include the indirect observation of $\rho (\omega )$. To this end, we introduce the vector $\vec {F} \in \mathbb {R}^{\tau _{\textrm{max}}}$ of components

137 $$\begin{aligned} F_t(\omega ) = \langle \mathcal {C}(t) \rho (\omega )\rangle = \int {\rm d}\vec {\eta }\, \mathcal {D}\rho \; \mathcal {C}(t) \, \rho (\omega )\, \mathbb {G}[\vec {\eta }, C_Y] \,\Pi [\rho ], \end{aligned}$$

together with the scalar $F_*$

138 $$\begin{aligned} F_*(\omega ) = \int \mathcal {D}\rho \; \rho (\omega )^2\; \Pi [\rho ] = \mathcal {K}^{\textrm{prior}}(\omega ,\omega ). \end{aligned}$$

The total covariance can be written as a block matrix

139 $$\begin{aligned} \Sigma ^{\textrm{tot}} = \begin{pmatrix} F_*(\omega ) & \vec {F}(\omega )^T \\ \vec {F}(\omega ) & \Sigma +C_Y \end{pmatrix}\;. \end{aligned}$$

Let $C^{\textrm{obs}}(t)$ be the correlator measured on the lattice by averaging over a gauge ensemble, and $C^{\, \mathrm{prior}}(t)$ the vector $\vec {\mathcal {C}}$ evaluated at $\rho = \rho ^{\textrm{prior}}$, whose components are

140 $$\begin{aligned} C^{\textrm{prior}}(t) = \mathcal {C}(t) \, |_{\rho ^{\textrm{prior}}} = \int \textrm{d}E \, b_T(t,E) \, \rho ^{\textrm{prior}}(E). \end{aligned}$$

The joint probability density for $\rho (\omega )$ and $\mathcal {C}(t)$ is also a Gaussian distribution

141 $$\begin{aligned} \mathbb {G} \left[ \rho - \rho ^{\textrm{prior}}, \, \vec {\mathcal {C}} - \vec {C}^{\; \mathrm{prior}} ; \, \Sigma ^{\textrm{tot}} \right] \;, \end{aligned}$$

and can be factorized as the product of the posterior probability density for $\rho (\omega )$ and the likelihood of the data. Both distributions are Gaussian, and therefore

142 $$\begin{aligned} \mathbb {G} \left[ \rho - \rho ^{\textrm{prior}}, \, \vec {\mathcal {C}} - \vec {C}^{\; \mathrm{prior}} ; \, \Sigma ^{\textrm{tot}} \right] = \mathbb {G} \left[ \rho - \rho ^{\textrm{post}}; \, \mathcal {K}^{\textrm{post}}\right] \; \mathbb {G} \left[ \vec {\mathcal {C}} - \vec {C}^{\; \mathrm{prior}} ; \, \Sigma + C_Y \right] . \end{aligned}$$

##### Exercise 5

Prove Eq. (142).

The posterior Gaussian distribution for the spectral density is centered around

143 $$\begin{aligned} \left. \rho ^{\textrm{post}}(\omega ) \right| _{\mathcal {C}=C^{\textrm{obs}}} = \rho ^{\textrm{prior}}(\omega ) + \vec {F}^T(\omega ) \frac{1}{\Sigma + C_Y} \left( \vec {C}^{\;\mathrm obs} - \vec {C}^{\; \mathrm{prior}} \right) , \end{aligned}$$

and has variance

144 $$\begin{aligned} \left. \mathcal {K}^\textrm{post}(\omega ,\omega ) \right| _{\mathcal {C}=C^{\textrm{obs}}} = \mathcal {K}^{\textrm{prior}}(\omega ,\omega ) - \vec {F}^T(\omega ) \frac{1}{\Sigma + C_Y} \vec {F}(\omega ). \end{aligned}$$

Note that the posterior mean obtained in Eq. 144 is a linear combination of the data, as in the Backus–Gilbert method discussed in the previous section. In order to explicitly make contact with the BG result that we obtained in the previous section, we introduce the coefficients

145 $$\begin{aligned} \vec {g}^{\mathrm{\, GP}} (\omega ) = \vec {F}^T \frac{1}{\Sigma + C_Y}, \end{aligned}$$

where the superscript GP reminds us that these are the coefficients obtained using the GP method.

Equation (145) shows that the problem is regularized by formulating it in terms of probability distributions. The numerical instability of the BG solution, which manifests itself in the very large coefficients $\vec {g}^{\mathrm{\, GP}} (\omega )$, is the consequence of the large condition number of the model covariance Σ of Eq. (136), which grows exponentially for acceptable choices of the model covariance $\mathcal {K}$. In the absence of errors on the data, the magnitude of the coefficients is determined by the inverse of the matrix Σ. For noisy data, the covariance $C_Y$ is added to the matrix Σ, thereby providing a cut-off for its lower modes. The magnitude of the coefficients is now determined by the condition number of $\Sigma + C_Y$. We will later show how the choice of $\mathcal {K}^{\textrm{prior}}$ can be used to tune the cut-off on the low modes of Σ.

Another important remark concerns the smearing kernel that one has implicitly introduced in Eq. (143)

146 $$\begin{aligned} \mathcal {S}^{\textrm{GP}}(\omega ,E) = \sum _{\tau =1}^{\tau _{\textrm{max}}} \vec {g}^{\mathrm{\, GP}} (\omega ) \,b_T(a\tau ,E). \end{aligned}$$

The center of the posterior distribution, $\rho ^{\textrm{post}}(\omega )$, is an estimator for a spectral density that is smeared with $\mathcal {S}^{\textrm{GP}}$. The unsmeared spectral density can be obtained if the prior is engineered, such that $\mathcal {S}^\textrm{GP}(\omega -E)$ is a Dirac delta. Such a distribution cannot be obtained by a finite linear combination of the regular functions $b_T(t,E)$, and one would need to extrapolate the result at vanishing smearing radius.

We conclude this section with a crucial remark on the nature of the prior. The functional behavior of the prediction is related to the prior through Eqs. (143) and (144). In particular, the covariance of the prior is known to affect the typical correlation length of the posterior. On the lattice, finite-volume spectral densities are targeted; therefore, this length should be smaller than the typical spacing between energy levels in order to capture the features of the underlying physics.

#### Choice of the priors

The prior model covariance used in this lectures is a variation of a Gaussian covariance

147 $$\begin{aligned} \mathcal {K}(\omega ,E) = \frac{e^{\alpha E}}{\lambda } \frac{e^{-\frac{(\omega -E)^2}{2\epsilon ^2}}}{\sqrt{2\pi } \epsilon } \equiv \frac{e^{\alpha E}}{\lambda } G_\epsilon (\omega -E), \end{aligned}$$

where ϵ, α, and λ are the hyperparameters that fully specify the prior. The motivations behind the choice made in Eq. (147) are the following. First, a Gaussian is a common choice in the literature [8, 13], with width ϵ and amplitude $\lambda ^{-1}$ as parameters to be chosen. In addition, the Gaussian distribution has a simple limit in which the covariance becomes diagonal by changing the parameter ϵ. The term $e^{\alpha E}$ allows us to control the deviations from the Gaussian case, and provides a link with the results obtained using the method of Ref. [9]. In order to set the notation, we specialize the previous equations to this choice of the prior

148 $$\begin{aligned} & \frac{\Sigma ^\epsilon _{tr}}{\lambda } = \int \textrm{d}E_1\, \textrm{d}E_2\; b_T(E_1,t) \, b_T(E_2,r)\, e^{\alpha E_1} \, \frac{G_\epsilon (E_1-E_2)}{\lambda }, \end{aligned}$$

149 $$\begin{aligned} & \frac{F^{\epsilon }_t(\omega )}{\lambda } = \int \textrm{d}E \, b_T(t,E) \, e^{\alpha E} \; \frac{G_\epsilon (E-\omega )}{\lambda }. \end{aligned}$$

The resulting expression for the coefficients of Eq. (145) is

150 $$\begin{aligned} \vec {g}^{\textrm{GP}}(\epsilon ; \omega ) = \vec {F}^{\, T}(\omega ) \, \frac{1}{\Sigma ^\epsilon + \lambda \, C_Y} \;. \end{aligned}$$

It is clear that λ is parametrizing the cut-off on the low modes of $\Sigma ^\epsilon$, thus introducing a bias. When λ becomes larger the role of the regularizing term, $C_Y$, is enhanced, and the coefficients $g_\tau$ become increasingly smaller. At smaller values of λ, the bias decreases, but it cannot be eliminated. Its dependence must be, therefore, addressed, and its effect quantified and controlled. The role of such parameter is extensively discussed in the context of Backus–Gilbert methods, and in particular in its formulation of Ref. [9], where it is usually prescribed to choose λ, such that the prediction for the spectral density is stable within statistical noise [14–16] upon variations of λ. From this “stability analysis”, the bias is assumed to be absorbed into the statistical error, a procedure that has been validated numerically; see for instance Refs. [14, 16, 17]. In the context of GPs, on the other hand, the hyperparameters (including λ) are selected, so that the resulting probability of observing the data

151 $$\begin{aligned} \mathbb {G} \left[ \vec {C}^{\, \mathrm obs} - \vec {C}^{\; \mathrm{prior}} ; \, \Sigma + C_Y \right] , \end{aligned}$$

is maximized [13, 18].^5^ Equivalently, one minimizes the “negative logarithmic likelihood” (NLL)

152 $$\begin{aligned} \frac{\tau _{\textrm{max}}}{2}\, \text {Log} (2\pi ) +\frac{1}{2} \, \text {Log}\, \text {det} \left( \Sigma + \text {Cov}_d \right) +\frac{1}{2} \, \left(\vec {C}^{\, \mathrm obs}- \vec {C}^{\; \mathrm{prior}}\right) \frac{1}{\Sigma +\text {Cov}_d} \left(\vec {C}^{\, \mathrm obs}- \vec {C}^{\; \mathrm{prior}}\right). \end{aligned}$$

**Fig. 4:**
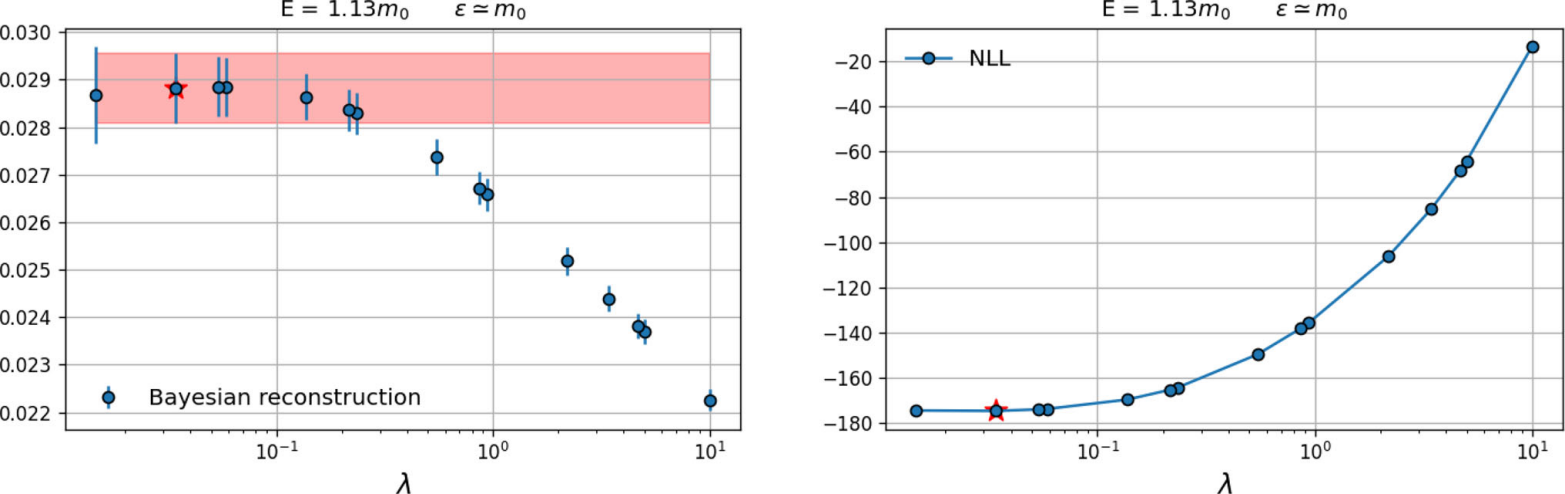
Combination of the stability analysis used (left) and a scan of the NLL (right). The smeared density shown in the left panel is obtained at a specific energy, and is evaluated from GPs, i.e., from Eqs. (143) and (144) for the central value and the error, respectively. The prior is the modified Gaussian of Eq. (147), with $\epsilon \simeq m_0$, the latter being the ground state of the channel. We only display value corresponding to α=0, because these correspond to a systematically smaller NLL. The purpose of this figure is to show that the treatment of the parameter λ from Ref. [9] and GPs can lead to compatible results. Details about the lattice data used for this example are found in the main text

In this setting, the fate of the bias introduced by λ could be considered opaque. We, therefore, assess whether porting the “stability analysis” that is carried in Ref. [9] into the Bayesian setup, can add insights in this regard. In Fig. 4, we show how the choice of λ affects the spectral reconstruction (left panel)^6^ at a specific energy. The corresponding values of the NLL are shown in the right panel of the same figure, with the minimum value highlighted. For large values of λ, the smeared spectral density changes considerably, showing a large dependence on the prior. The corresponding values of the NLL are large. Remarkably, as the NLL approaches its minimum, the dependence on the prior softens. The horizontal band in the left panel of Fig. 4 is obtained at the value of λ that minimizes the NLL, which is flagged by a star in the right panel. This result suggests that the treatment of the bias might not be drastically different in these two cases, despite the very different approaches.

**Fig. 5:**
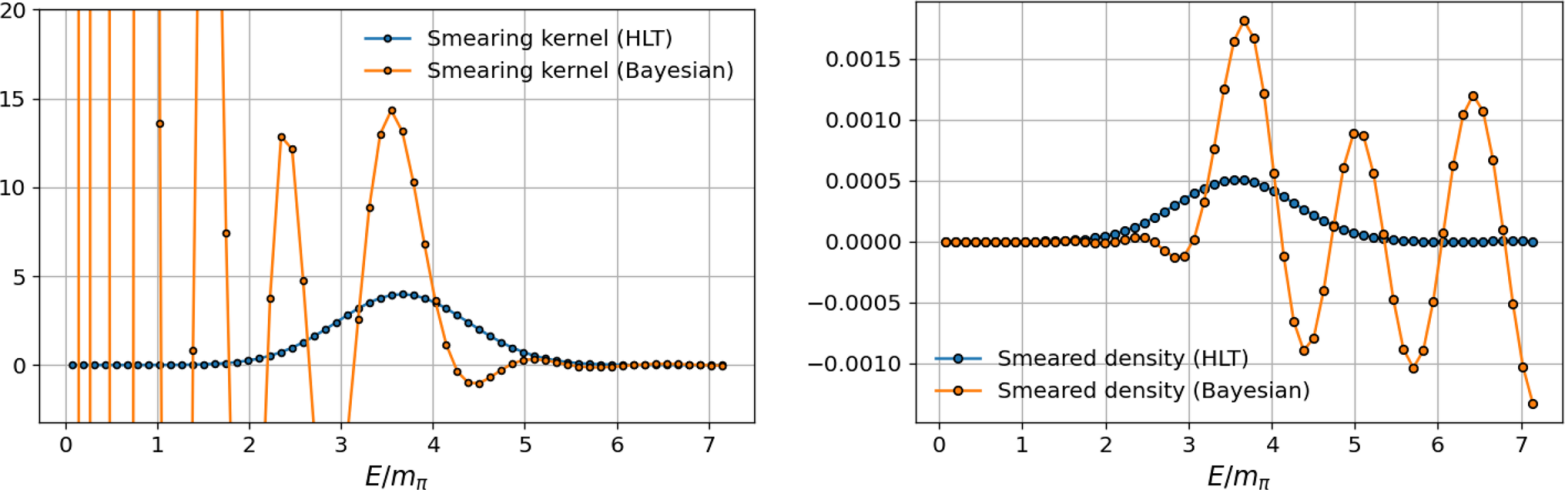
Left panel: examples of the function smearing the spectral density at the energy $E = 3.7 m_\pi$ in the Bayesian setup (orange) and from the HLT procedure (blue) using exact data. The latter targets a Gaussian kernel with a width of approximately 0.75 in units of $m_\pi$, which is reconstructed with great precision given the lack of uncertainties on the input data. For the Bayesian calculation, we use the same Gaussian function as a prior, but we have less control on the output function, which in this case features oscillations with a length scale determined by the prior. The right panel displays the reconstructed smeared spectral densities from the same data. This example uses $t_{\textrm{max}}=32$ data points

Another important remark, already stated in the previous section, is that the value obtained at the minimum of the NLL (horizontal band in Fig. 4) has to be interpreted and understood as a spectral density that is smeared with the appropriate smearing kernel, which is not known a priori in the Bayesian formulation given in this section. In this respect, this method is closer to the original proposal of Backus and Gilbert rather than the modification of Ref. [9]. For this reason, we find it instructive to show, in the left panel of Fig. 5, an example of the smearing kernel at a specific energy ($\omega = 3.7 m_\pi$) obtained with the Bayesian setup (orange), as opposed to the smearing kernel obtained from the method (in blue) of Ref. [9] (HLT in short) that will be described in the next section. The Bayesian kernel is not known a priori: its behavior is constrained by the covariance of the prior, which can produce a smoother or more rapidly changing function. In the example of Fig. 5, the prior is defined in Eq. 147, with $\epsilon = 0.75 m_\pi$. The output thus features, around $\omega = 3.7 m_\pi$, oscillation that have roughly a wavelength of ϵ. In the right panel of the same figure, the smeared spectral density obtained with the two methods is also displayed. In this example, we have used synthetic data without any statistical noise, in order to showcase what each method does in the ideal limit of exact data. The input correlator contains a single state with $E/m_\pi = 3.7$.

## Neural networks

### Neural networks basics

In the context of PDF fitting, the NNPDF collaboration has pioneered the use of neural networks as a flexible parametrization of PDFs, which allows to reduce the bias introduced by the choice of a specific functional form. The NNPDF approach is based on the idea of using a large number of parameters (the weights and biases of the neural network) to fit the data, while monitoring the fitting process in order to prevent overfitting, see e.g. Ref. [2] for details. In this language, the ensemble of NN replicas at initialization defines the prior probability distribution of the model, and the training process is used to update the probability distribution in light of the data, thus defining the posterior distribution. Note that, while we have prior and posterior distributions, the inference is not performed according to Bayes’ theorem.

In these section, we briefly recall some basic notions about neural networks, and develop a statistical field theory description of the behavior of neural networks at initialization, which will be the foundation for the discussion of the training process in the next section.

A neural network is one way of parametrizing a function, *f*(*x*), using interconnected layers of computing units, which are called *neurons* by analogy with the biological neurons. We are going to focus here on the simplest type of neural network, which is the feed-forward neural network, where the information flows in one direction from the input layer, identified by ℓ=0 to the output layer, identified by ℓ=L. The neuron in each layer is labeled by an index *i*, and the number of neurons in layer ℓ is denoted by $n_\ell$. The input of the network is *x*, which is a vector of dimension $n_0$, and the output is *f*(*x*), which is a vector of dimension $n_L$. If we are parametrizing a function that returns a scalar, then $n_L=1$. At each neuron, we associated a so-called *pre-activation* function, $\phi ^{(\ell )}_i(x)$. The indices ℓ and *i* identify the neuron, and we explicitly wrote the dependence on the input *x*. The output of the neuron is obtained by applying the *activation function* ρ to the pre-activation

153 $$\begin{aligned} \rho ^{(\ell )}_i(x) = \rho \left( \phi ^{(\ell )}_i(x) \right) . \end{aligned}$$

The pre-activation function are recursively defined

154 $$\begin{aligned} \phi ^{(\ell )}_i(x) = \sum _{j=1}^{n_{\ell -1}} W^{(\ell )}_{ij} \, \rho ^{(\ell -1)}_j(x) + b^{(\ell )}_i, \end{aligned}$$

with $\phi ^{(0)}_i(x) = x_i$. The $W^{(\ell )}$ are the *weight* matrices, $b^{(l)}$ are the *bias* vectors. For each input *x*, the recursion relations above allow us to compute the pre-activation and the output of each neuron, and ultimately the output of the network, $f_i(x)=\phi ^{(L)}_i(x)$.

The weights and biases are the parameters of the neural network, they are initialized according to some prescription and they are then determined by the training process. Neural networks provide a flexible parametrization of functions and are known to be universal approximators for sufficiently large number of parameters. They were originally introduced for PDF fitting precisely in order to avoid any bias introduced by a fixed functional form with insufficient degrees of freedom.

Typically, we consider a discrete set of input points, labeled by an index α as discussed in the previous sections. The input points are therefore $x_\alpha$. In order to simplify the notation, we introduce

155 $$\begin{aligned} \phi ^{(\ell )}_{i\alpha } = \phi ^{(\ell )}_i(x_\alpha ), \qquad \rho ^{(\ell )}_{i\alpha } = \rho ^{(\ell )}_i(x_\alpha ). \end{aligned}$$

**Fig. 6:**
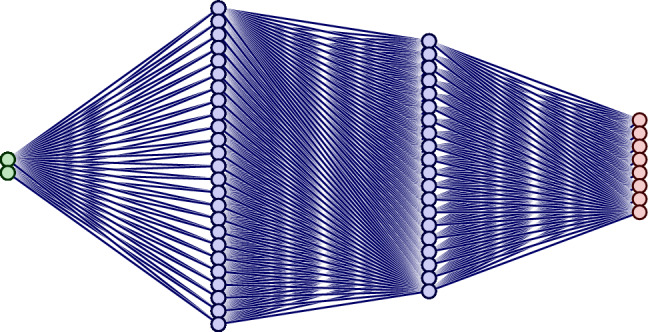
A feed-forward neural network with L=3 layers and $n_0=2$, $n_1=25$, $n_2=20$, $n_3=8$. The input layer is in green on the left, the output layer is in red on the right, and the hidden layers are in blue. This is the actual architecture of the neural network used by the NNPDF collaboration for PDF fitting; see Ref. [2]

It is important to distinguish between the index α, which labels the input points, and the index *i*, which labels the neurons. For each α, the input *x* could be a vector, whose components are labeled by the index $i=1, \ldots , n_0$. A typical example of an NN is shown in Fig. 6. Each dot in the figure represents a neuron, so that for each dot, we have a pre-activation $\phi ^{(\ell )}_{i\alpha }$ and an output $\rho ^{(\ell )}_{i\alpha }$. The actual values of the pre-activation and the output depend on the input $x_\alpha$, which is not explicitly shown in the figure. The connections between the dots represent the weights $W^{(\ell )}_{ij}$, and the bias $b^{(\ell )}_i$ is associated with each neuron. The input layer is in green on the left, the output layer is in red on the right, and the hidden layers are in blue. Note that the NN shown in the figure has two neurons in the input layer. Having two neurons in the input layer allows us to use both *x* and logx as inputs, which enhances the sensitivity of the NN to the small-*x* region.

### Neural networks at initialization

The weights and biases of the NN are initialized as independent Gaussian variables

156 $$\begin{aligned} W^{(\ell )}_{ij} \sim \mathcal {N}\left( 0, \frac{C_W}{n_{\ell -1 }}\right) , \qquad b^{(\ell )}_i \sim \mathcal {N}(0, C_b). \end{aligned}$$

Both distributions have zero mean. The variance of the weights in layer ℓ is scaled by the number of neurons in the previous layer, $n_{\ell -1}$. This is a common choice, sometimes referred to as the Glorot initialization [19]. By inspecting the recursion relation in Eq. 154, it is clear that the scaling of the variance guarantees that the pre-activations have a finite limit when the number of neurons in the previous layer becomes large. The average over the probability distributions in Eq. 156 yields

157 $$\begin{aligned}&\mathbb {E}[W^{(\ell )}_{ij}] = 0, \end{aligned}$$

158 $$\begin{aligned}&\mathbb {E}[b^{(\ell )}_i] = 0, \end{aligned}$$

159 $$\begin{aligned}&\mathbb {E}[W^{(\ell )}_{ij} W^{(\ell )}_{i'j'}] = \delta _{ii'}\delta _{jj'} \frac{C_W}{n_{\ell -1}}, \end{aligned}$$

160 $$\begin{aligned}&\mathbb {E}[b^{(\ell )}_i b^{(\ell )}_{i'}] = \delta _{ii'} \, C_b, \end{aligned}$$

and all other correlations—in particular correlations between different layers—vanish. The $\phi ^{(0)}$ are given by the values of the input, and therefore are deterministic quantities. All other pre-activations are stochastic variables and we are interested in their joint probability distribution

161 $$\begin{aligned} P\left( \phi ^{(L)} \ldots \phi ^{(1)}\right) = P\left( \phi ^{(L)}|\phi ^{(L-1)}\right) \ldots P\left( \phi ^{(1)}|\phi ^{(0)}\right) . \end{aligned}$$

The following derivations are based on the recent review in Ref. [20].

***A forward recursion relation.***

The probability distribution of the pre-activations in layer ℓ obeys a forward recursion relation, which allows to compute it from the probability distribution of the pre-activations in layer ℓ-1

162 $$\begin{aligned} P\left( \phi ^{(\ell +1)} | \phi ^{(0)}\right) = \int \textrm{d}\phi ^{(\ell )} \, P\left( \phi ^{(\ell +1)} | \phi ^{(\ell )}\right) P\left( \phi ^{(\ell )} | \phi ^{(0)}\right) . \end{aligned}$$

The conditional probability distribution $P\left( \phi ^{(\ell +1)} | \phi ^{(\ell )}\right)$ can be computed by integrating over the weights and biases

163 $$\begin{aligned} P\left( \phi ^{(\ell +1)}|\phi ^{(\ell )}\right)&= \int \prod _{i,j} \textrm{d}W^{(\ell +1)}_{ij} \prod _i \textrm{d}b^{(\ell +1)}_i \, p\left( W^{(\ell +1)}\right) p\left( b^{(\ell +1)}\right) \, \nonumber \\&\qquad \times \ \prod _i \delta \left( \phi ^{(\ell +1)}_i - \sum _j W^{(\ell +1)}_{ij} \rho ^{(\ell )}_j - b^{(\ell +1)}_i \right) , \end{aligned}$$

where $p\left( W^{(\ell +1)}\right)$ and $p\left( b^{(\ell +1)}\right)$ are the probability distributions of the weights and biases in layer ℓ+1 given in Eq. 156. The delta functions in the last line enforce the definition of the pre-activations in terms of the weights, biases, and activations in the previous layer. For fixed values of the pre-activations in layer ℓ, the pre-activations in layer ℓ+1 are linear combinations of the weights and biases, and therefore, their probability distribution is Gaussian

164 $$\begin{aligned} P\left( \phi ^{(\ell +1)}|\phi ^{(\ell )}\right) = \frac{1}{\left| 2\pi \hat{G}^{(\ell +1)}\right| ^{n_{\ell +1}/2}} \, \exp \left( -\frac{1}{2} \phi ^{(\ell +1)}_{\alpha _1} \cdot \phi ^{(\ell +1)}_{\alpha _2} \left( \hat{G}^{(\ell +1)}\right) ^{-1}_{\alpha _1 \alpha _2} \right) , \end{aligned}$$

where the scalar product indicates the contraction of the neuron indices, and we introduced the *propagator*

165 $$\begin{aligned} \hat{G}^{(\ell +1)}_{\alpha _1 \alpha _2} = C_b + C_W \, \frac{1}{n_\ell } \rho ^{(\ell )}_{\alpha _1} \cdot \rho ^{(\ell )}_{\alpha _2}. \end{aligned}$$

The factor of $1/n_\ell$ in Eq. 165 comes from the scaling of the variance of the weights in Eq. 156. The scalar product between the outputs $\rho ^{(\ell )}$ involves a sum over the neuron indices, and therefore, it is of order $n_\ell$; the propagator has a finite limit when the number of neurons in layer ℓ becomes large. Clearly, $\hat{G}^{(\ell +1)}$ depends on the pre-activations $\phi ^{(\ell )}$.

#### Exercise 6

Show that the correlators of the pre-activations in any layer ℓ are symmetric under rotations in the space of neurons, i.e.

166 $$\begin{aligned} \mathbb {E}\left[ R_{i_1j_1} \phi ^{(\ell )}_{j_1 \alpha _1} \ldots R_{i_nj_n} \phi ^{(\ell )}_{j_n \alpha _n} \right] = \mathbb {E}\left[ \phi ^{(\ell )}_{i_1 \alpha _1} \ldots \phi ^{(\ell )}_{i_n \alpha _n} \right] , \end{aligned}$$

where *R* is an orthogonal matrix in $\text {SO}(n_{\ell })$. Equation(166) implies that the probability distribution is also invariant under rotations, and therefore, it can only be a function of $\text {SO}(n_{\ell })$ invariants, i.e. it can only depend on the scalar product of the pre-activations.

In the first hidden layer, ℓ=1, the pre-activations are linear combinations of the weights and biases

167 $$\begin{aligned} \phi ^{(1)}_{i\alpha } = \sum _j W^{(1)}_{ij} \rho \left( x_{\alpha j}\right) + b^{(1)}_i, \end{aligned}$$

and therefore, they are Gaussian variables, with zero mean and covariance

168 $$\begin{aligned} \mathbb {E}\left[ \phi ^{(1)}_{i\alpha } \phi ^{(1)}_{i'\alpha '} \right] = \delta _{ii'} \left( C_b + C_W \, \frac{1}{n_0} \sum _j \rho \left( x_{\alpha j}\right) \rho \left( x_{\alpha ' j}\right) \right) . \end{aligned}$$

One can readily check, by writing down the explicit expression, that the distributions are no longer Gaussian as soon as we move to ℓ>1.

***Effective theory approach.***

It is useful to rewrite the recursion relation in (162) by introducing an effective action, $S^{(\ell )}$, defined by

169 $$\begin{aligned} P\left( \phi ^{(\ell )} | \phi ^{(0)}\right) = \frac{1}{Z^{(\ell )}} \, e^{-S^{(\ell )}(\phi ^{(\ell )})}, \end{aligned}$$

where $Z^{(\ell )}$ is a normalization factor. The recursion relation in Eq. 162 can be rewritten as

170 $$\begin{aligned} \frac{e^{-S^{(\ell +1)}(\phi ^{(\ell +1)})}}{Z^{(\ell +1)}} = \int \textrm{d}\phi ^{(\ell )} \, \frac{e^{-S^{(\ell )}(\phi ^{(\ell )})}}{Z^{(\ell )}} \, \frac{\exp \left( -\frac{1}{2} \phi ^{(\ell +1)}_{\alpha _1} \cdot \phi ^{(\ell +1)}_{\alpha _2} \left( \hat{G}^{(\ell +1)}\right) ^{-1}_{\alpha _1 \alpha _2} \right) }{\left| 2\pi \hat{G}^{(\ell +1)}\right| ^{n_{\ell +1}/2}}. \end{aligned}$$

In order to extract further information from the recursion relation, we need to parametrize the effective action. The most general form of the effective action is

171 $$\begin{aligned} S^{(\ell )}(\phi ^{(\ell )}) = \frac{1}{2}\, \gamma ^{(\ell )}_{\alpha _1 \alpha _2} \, \left( \phi ^{(\ell )}_{\alpha _1} \cdot \phi ^{(\ell )}_{\alpha _2}\right) + \frac{1}{8 n_{\ell -1}} \, \gamma ^{(\ell )}_{\alpha _1 \alpha _2; \alpha _3 \alpha _4} \, \left( \phi ^{(\ell )}_{\alpha _1} \cdot \phi ^{(\ell )}_{\alpha _2} \right) \left( \phi ^{(\ell )}_{\alpha _3} \cdot \phi ^{(\ell )}_{\alpha _4} \right) + \cdots , \end{aligned}$$

where we introduced the *couplings* $\gamma ^{(\ell )}_{\alpha _1 \alpha _2}$ and $\gamma ^{(\ell )}_{\alpha _1 \alpha _2; \alpha _3 \alpha _4}$, which are functions of the layer ℓ and of the input points. Note that because of symmetry reasons, the effective action can only depend on the scalar product of the pre-activations. The factors in the action reflect the symmetries of each term, and the scaling with the number of neurons in the previous layer is chosen, so that the couplings have a finite limit when $n_{\ell -1}$ becomes large. We have omitted higher order terms in the effective action; they involve higher powers of the scalar product of the pre-activations, and they are suppressed by higher powers of $1/n_{\ell -1}$.

At leading order, the second and fourth cumulants are respectively

172 $$\begin{aligned}&\langle \phi ^{(\ell )}_{i_1,\alpha _1} \phi ^{(\ell )}_{i_2,\alpha _2}\rangle = \delta _{i_1 i_2} K^{(\ell )}_{\alpha _1\alpha _2} + O(1/n_{\ell -1}), \end{aligned}$$

173 $$\begin{aligned}&\langle \phi ^{(\ell )}_{i_1,\alpha _1} \phi ^{(\ell )}_{i_2,\alpha _2} \phi ^{(\ell )}_{i_3,\alpha _3} \phi ^{(\ell )}_{i_4,\alpha _4}\rangle _c = O(1/n_{\ell -1}), \end{aligned}$$

where^7^

174 $$\begin{aligned} K^{(\ell )}_{\alpha _1\alpha _2} = \left( \gamma ^{(\ell )}\right) ^{-1}_{\alpha _1\alpha _2}. \end{aligned}$$

#### Exercise 7

Derive the Feynman rules for the effective theory defined by the action in Eq. (171), and use them to compute the second and fourth cumulants of the pre-activations at leading order in $1/n_{\ell -1}$, thus showing that they are given by the expressions in Eqs. (172) and (173).

The “evolution” of the couplings as we go deep in the NN, i.e., the dependence of the couplings on ℓ, is governed by Renormalization Group (RG) equations, which preserve the power counting in powers of $1/n_{\ell }$. At leading order

175 $$\begin{aligned} K^{(\ell +1)}_{\alpha _1\alpha _2}&= \left. C_b^{(\ell +1)} + C_w^{(\ell +1)} \frac{n_\ell }{n_\ell +n_{\ell +1}}\frac{1}{n_\ell } \langle \vec {\rho }^{\,(\ell )}_{\alpha _1} \cdot \vec {\rho }^{\,(\ell )}_{\alpha _2} \rangle \right| _{O(1)} \end{aligned}$$

176 $$\begin{aligned}&= C_b^{(\ell +1)} + C_w^{(\ell +1)} \frac{n_\ell }{n_\ell +n_{\ell +1}}\frac{1}{n_\ell } \langle \vec {\rho }^{\,(\ell )}_{\alpha _1} \cdot \vec {\rho }^{\,(\ell )}_{\alpha _2} \rangle _{K^{(\ell )}}, \end{aligned}$$

where

$$\begin{aligned} \frac{1}{n_\ell } \langle \vec {\rho }^{\,(\ell )}_{\alpha _1} \cdot \vec {\rho }^{\,(\ell )}_{\alpha _2} \rangle _{K^{(\ell )}} = \int \mathcal {D}\phi \, \frac{e^{-\frac{1}{2} \left( K^{(\ell )}\right) ^{-1}_{\beta _1\beta _2} \phi _{\beta _1} \phi _{\beta _2}}}{\left| 2\pi K^{(\ell )}\right| ^{1/2}}\, \rho (\phi _{\alpha _1}) \rho (\phi _{\alpha _2}), \end{aligned}$$

and

177 $$\begin{aligned} \mathcal {D}\phi = \prod _{\alpha =1}^{N_{\textrm{grid}}} \textrm{d}\phi _\alpha . \end{aligned}$$

**Fig. 7:**
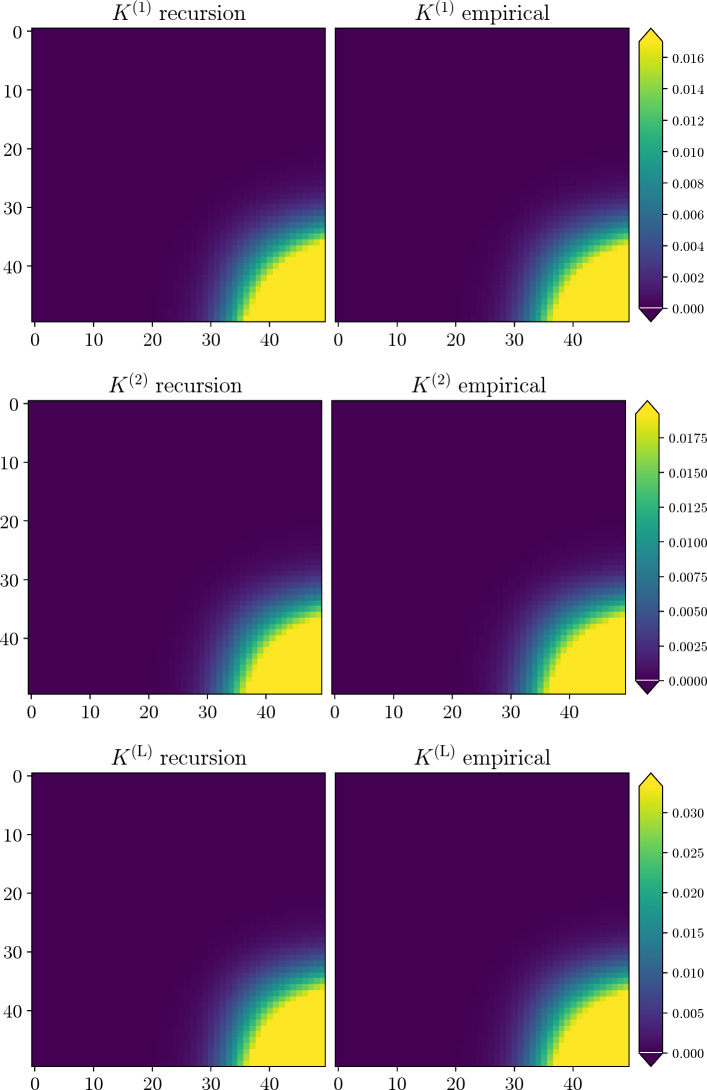
The empirical (left) and analytical (right) covariance matrices of the first, second, and output layers of the NNPDF architecture (top to bottom). The covariance in the left panel is computed “bootstrapping” over an ensemble of replicas, initialized using the Glorot normal distribution. The covariance in the right panel is obtained by solving Eq. (176) numerically. In order to reduce the bootstrap errors in the empirical covariance, an ensemble of 1000 replicas has been used for this figure

**Fig. 8:**
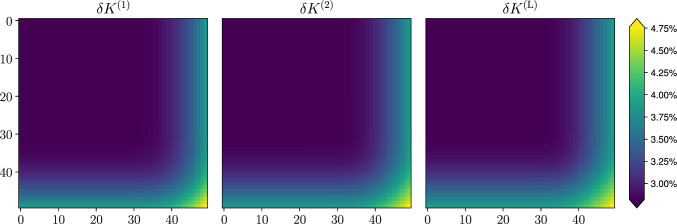
Relative difference between the empirical kernel, computed from an ensemble of networks at initialization, and the recursive kernel obtained by iterating Eq. (176) for the three layers of the NNPDF architecture. An ensemble of 1000 replicas has been used to reduce the bootstrap errors in the empirical covariance

Note that the integration variables in Eq. (177) do not have a neuron index and the integrals are $N_{\textrm{grid}}$ dimensional integrals. Equation (176) is iterated for the NNPDF architecture, yielding $K^{(\ell )}$ for arbitrary ℓ, i.e., the covariance at initialization for various depths. These are compared with the empirical covariance computed from an ensemble replicas in Fig. 7 for the first two hidden layers and the output layer. Furthermore, the relative difference between the empirical covariance and the theoretical prediction is shown in Fig. 8. In order to reduce the bootstrap errors in the empirical covariance, an ensemble with $N_{{\textrm{rep}}}=1000$ has been used for these figures. The agreement between the theoretical prediction and the empirical computation is excellent, confirming the validity of the large-network expansion even for networks of moderate size, as those used in the NNPDF fits.

**Fig. 9:**
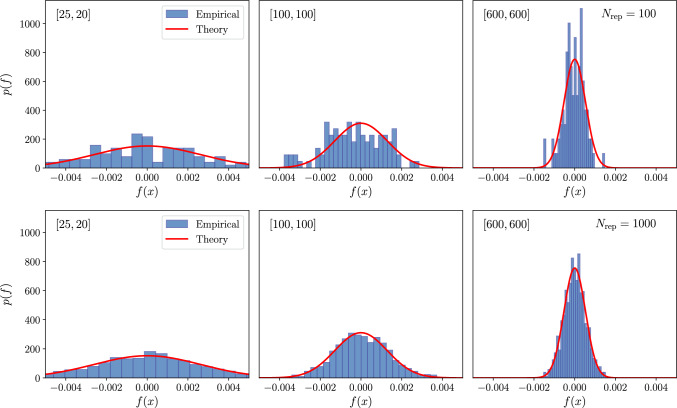
Sampled distribution of the output $xT_3$ at x=0.0065 for two different ensemble sizes, $N_{{\textrm{rep}}}=100$ (top) and $N_{{\textrm{rep}}}=1000$ (bottom). Each column shows the distribution for a different network architecture; the latter displayed in the top left corner of each panel. The red line represents the predicted Gaussian distribution as dictated by the kernel recursion formula in Eq. (176)

**Fig. 10:**
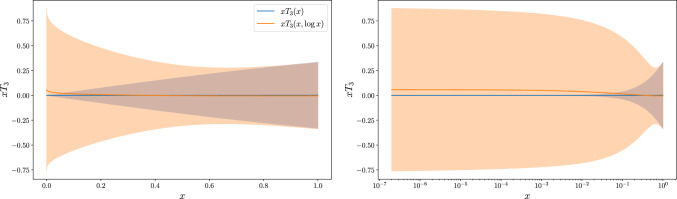
The output of the ensemble of neural networks at initialization using the NNPDF architecture in linear (left) and logarithm (right) scale. We compare the case of linear input *f*(*x*) (blue) and the case of scaled input f(x,logx) (orange). The solid lines represent the mean value computed over an ensemble of 100 replicas, while the shaded bands represent the one-sigma uncertainty computed as the variance over the same ensemble. In the figure, we show $xT_3$ as used in the following sections

As a consequence of the symmetry of the probability distribution, the mean value of the fields at initialization needs to vanish, while their variance at each point $x_\alpha$ is given by the diagonal matrix elements of $K^{(\ell )}$. In Fig. 9, the expected distribution is compared against the empirical distribution of output fields for a selected value of *x*, using two ensembles of replicas with $N_{{\textrm{rep}}}=100$ and $N_{{\textrm{rep}}}=1000$, respectively. Inspecting the figures, we conclude that the recursion formula, Eq. (176), accurately reproduces the output distribution of the NNPDF networks at initialization, provided that a sufficiently large ensemble of replicas is used to sample the distribution. Finally, Fig. 10 shows the mean and variance of the output at initialization across all values of *x* for an ensemble of $N_{{\textrm{rep}}}=100$ neural networks generated using the NNPDF architecture. We compare two cases: linear input *f*(*x*) and scaled input f(x,logx). The central value is computed as the average over replicas

178 $$\begin{aligned} \bar{f}_{i\alpha } = \bar{f}_{i}(x_\alpha ) = \frac{1}{N_{{\textrm{rep}}}} \sum _{k=1}^{N_{{\textrm{rep}}}} f^{(k)}_i(x_\alpha ), \end{aligned}$$

and, likewise, the variance $\sigma ^2_{i\alpha }$ is computed over the same ensemble of replicas

179 $$\begin{aligned} \sigma ^2_{i\alpha } = \frac{1}{N_{{\textrm{rep}}}-1} \sum _{k=1}^{N_{{\textrm{rep}}}} \left( f^{(k)}_i(x_\alpha ) - \bar{f}_{i}(x_\alpha )\right) ^2. \end{aligned}$$

As is clear from the figure, the choice of input scaling has a significant impact on the prior uncertainty, especially in the small-*x* region. In the following, we neglect this effect and focus on the case of linear input *f*(*x*).

## Training neural networks

We have summarized some properties of the neural network at initialization, showing that the normal Glorot initialization leads to a prior distribution that is a Gaussian processes with small corrections. In the final part of these lectures, we analyze the training dynamics of the neural network, with the aim of understanding how the process of training yields the posterior distribution of the solution to the inverse problem. We follow very closely the presentation of Ref. [21].

In the context of machine learning, specifically when dealing with neural networks, optimization is an iterative algorithm that updates the parameters of the network in order to minimize a figure of merit defined appropriately. The direction toward the minimum is defined by the gradient of the figure of merit. Due to the large number of parameters that characterize a neural network, and the complex functional form induced by the recursive definition of the network, the figure of merit (also known as *error function*, *loss function*, or simply *loss*) is a non-convex high-dimensional function of the parameters, leading to numerical challenges in the minimization task. In addition, in order to avoid *over-* and *under-learning*, training algorithms require a *stopping criterion*, which specifies the optimal condition to end the training process. While these algorithms have achieved remarkable empirical success, a theoretical understanding of the optimization process remains elusive. In these lectures, we want to focus on understanding the dynamics, without being bogged down by unnecessary technical details; therefore, we work with the simplest gradient method, i.e., Gradient Descent (GD).

We work out in detail one specific example, in the context of PDF determinations, where we consider a reduced dataset for which predictions can be computed using just one flavor combination of the PDFs, so that the theoretical prediction for the data remains simply

180 $$\begin{aligned} T_I[f] = \int \textrm{d}x\, C_{I}(x) f(x). \end{aligned}$$

The details of the dataset and the definition of the flavor combination are not relevant for these lectures, and the interested reader can find more details in Ref. [21].

### Training in functional space

For analytical tractability, GD is described as a continuous flow of the parameters θ in training time *t* along the negative gradient of the loss function $\mathcal {L}$. For sufficiently small learning rates η, this continuous flow approximates the discrete GD trajectory in parameter space, as extensively discussed in Ref. [22]. The continuous Gradient Flow (GF) is then given by

181 $$\begin{aligned} \frac{\textrm{d}}{\textrm{d}t}&\theta _{t,\mu } = -\nabla _\mu \mathcal {L}_t, \end{aligned}$$

where $\theta _{t,\mu }$ and $\mathcal {L}_t$ identify, respectively, the parameters and the loss function at training time *t*. We distinguish between the continuous training time *t* and the discrete epochs of GD, the latter denoted using the capital letter *T*. For the purpose of this lecture, the two are related through a constant learning rate, t=ηT with $\eta = 10^{-5}$, and we will use them interchangeably.

We focus here on quadratic loss functions that are obtained as the negative logarithm of Gaussian data distributions around their theoretical predictions; using the notation introduced in Sect. 2, the loss function is given by

182 $$\begin{aligned} \mathcal {L}_t = \frac{1}{2} \left( Y - T[f_t]\right) ^T C_Y^{-1} \left( Y - T[f_t]\right) , \end{aligned}$$

where $f_t$ is the output of the network at training time *t*, obtained from the time-dependence of the internal parameters, and $C_Y$ is the covariance of the data. Note that the loss function at training time *t* is computed using the theoretical prediction $T[f_t]$, computed using a grid of points for $f_t$ at training time *t*. For a quadratic loss, the gradient is

183 $$\begin{aligned} \nabla _\mu \mathcal {L}_t = - \left( \nabla _\mu f_t\right) ^T \left( \frac{\partial T}{\partial f}\right) _t C_Y^{-1} \epsilon _t, \end{aligned}$$

where writing explicitly the data index

184 $$\begin{aligned} \epsilon _{t,I} = Y_I - T_I[f_t], \quad I=1, \ldots , N_{\textrm{dat}}. \end{aligned}$$

For the specific case of a quadratic loss function, the gradient is proportional to $\epsilon _t$, which is the difference between the theoretical prediction and the data at training time *t*. If at some point during the training the theoretical predictions reproduce all the data, the training process ends.

A further simplification is obtained in the case of data that depend linearly on the unknown function *f*. In this case, the Jacobian of the theoretical prediction with respect to the fields is independent of the training time

185 $$\begin{aligned} \left( \frac{\partial T_I}{\partial f_{i\alpha }}\right) _t = (\textrm{FK})_{Ii\alpha }. \end{aligned}$$

A few algebraic steps allow the flow of parameters θ to be translated into a flow for the fields

186 $$\begin{aligned} \frac{\textrm{d}}{\textrm{d}t}&f_{t,i_1\alpha _1} = (\nabla _\mu f_{t,i_1\alpha _1}) \frac{\textrm{d}}{\textrm{d}t}\theta _\mu = \Theta _{t,i_1\alpha _1i_2\alpha _2} (\textrm{FK})^T_{i_2\alpha _2I} \left( C_Y^{-1}\right) _{IJ} \epsilon _{t,J}, \end{aligned}$$

where we have defined the Neural Tangent Kernel [23]

187 $$\begin{aligned} \Theta _{t,i_1\alpha _1i_2\alpha _2} = \sum _\mu \nabla _\mu f_{t,i_1\alpha _1} \nabla _\mu f_{t,i_2\alpha _2}. \end{aligned}$$

If we introduce the matrix

188 $$\begin{aligned} \left( J_t\right) _{i\alpha ,\mu } = \nabla _\mu f_{t,i\alpha }, \end{aligned}$$

the NTK can be written as $\Theta _t = J_t J_t^T$. The NTK is a positive-semidefinite matrix that encodes the dependence of the training dynamics on the architecture of the network. The flow equation, Eq. (186), is a non-linear integro-differential equation that describes the evolution of the fields during training.

In order to facilitate the discussion in Sect. 7.1.1, Eq. (186) can be rewritten in a more compact form. We first omit the indices and write for instance

189 $$\begin{aligned} \left( \frac{\partial T}{\partial f}\right) _t = (\textrm{FK}), \quad \Theta _t = \left( \nabla _\mu f_t\right) \left( \nabla _\mu f_t\right) ^T. \end{aligned}$$

Then, using the definition of the error in Eq. (184), we can rewrite Eq. (186) as

190 $$\begin{aligned} \frac{\textrm{d}}{\textrm{d}t}f_t = -\Theta _t M f_t + b_t, \end{aligned}$$

where

191 $$\begin{aligned} M&= (\textrm{FK})^TC_Y^{-1} (\textrm{FK}), \quad b_t = \Theta _t (\textrm{FK})^TC_Y^{-1} Y. \end{aligned}$$

#### Exercise 8

Derive the flow equation, Eq. (190), starting from the continuous Gradient Flow equation.

Here, *M* is a positive-semidefinite matrix that depends only on the data covariance and the FK tables that enter the theoretical predictions, while *b* is a vector that depends (among other quantities) on the central value of the data. Note that any vector *f* that is in the kernel of $(\textrm{FK})$ is necessarily in the kernel of *M*, kerM. In turn, the vectors in kerM do not contribute to the flow evolution, as seen explicitly in Eq. (190).

There are two important points that we want to emphasize. First, although derived in the context of neural networks, these equations do not refer to a specific parametrization. Indeed, these remain valid even when an explicit functional form is chosen to parametrize the unknown function *f*. Second, note that the flow equation, Eq. (190), depends on two matrices, Θ and *M*. The former encodes the model dependence, while the latter contains the physical information. The interplay between these two matrices is crucial for understanding the training dynamics, as discussed in Sect. 6.2.3. Finally, the NTK derived in Eq. (187) is inherently time-dependent in a complex way, which precludes any attempt at integrating Eq. (190) analytically. We come back to this point in Sect. 7.1.1, after discussing the properties of the NTK during training.

### Inside the training dynamics: an NTK perspective

From Eqs. (187) and (190), we observe that the NTK encodes the dependence on the architecture of the network and governs its training dynamics. The analysis of the NTK properties is one way to unravel the behavior of the network during training.

#### NTK at initialization

Before training, the NTK is blind to data and depends on the *x*-grid of input and on the architecture, as shown in Eq. (187). The NTK is a function of the fields *f*, which are stochastic variables described by their joint probability distribution. Therefore, the NTK is also a stochastic variable, with its own probability distribution, which we represent as usual as a set of replicas.

**Fig. 11:**
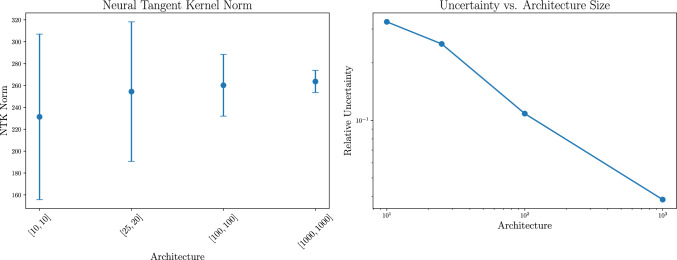
Frobenius norm of the NTK at initialization, $\Vert \Theta _0 \Vert$, as a function of the width of the network. On the left, the central values and uncertainty bands are obtained as the mean and one-sigma deviation of the ensemble of networks. The labels on the horizontal axis indicate the respective widths of the two hidden layers in the networks considered. The plot on the right shows the relative uncertainty. It is interesting to note the decrease of the relative uncertainty as the architecture of the network is increased. For larger networks, the sensitivity to a change of the network parameters fluctuates less

In the large-width limit, the variance of the NTK over the set of replicas is expected to go to zero with the width of the hidden layers. In order to quantify the variation of the NTK, we start by computing the Frobenius norm of the NTK over an ensemble of networks for different architectures. For each architecture, we consider the standard deviation of the norm as a statistical estimator of the variations of the NTK. The result is displayed in Fig. 11. Even though the Frobenius norm is a coarse indicator of the variations of the NTK, the figure shows clearly that the variance of the norm becomes smaller with the size of the network, which is consistent with the theoretical expectation that the NTK should not fluctuate for infinite-width networks.^8^

**Fig. 12:**
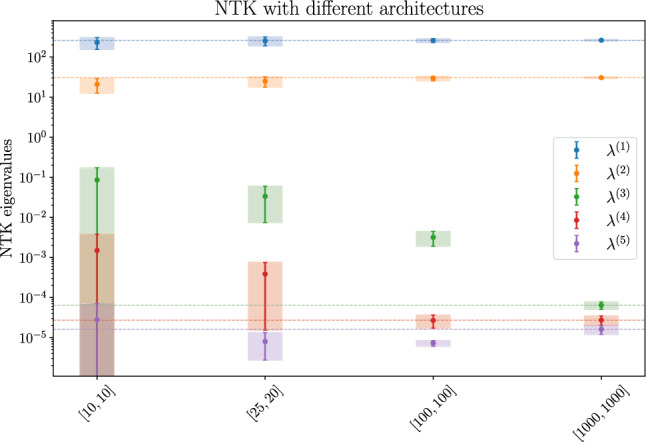
Spectrum of the NTK at initialization for the architectures shown in Fig. 11. The labels on the horizontal axis indicate the respective widths of the two hidden layers in the networks considered. Error bands correspond to one-sigma uncertainties over the ensemble of networks. The hierarchy of the eigenvalues is independent of the size of the network. In agreement with the data in Fig. 11, the fluctuations of the eigenvalues decrease as the width of the layers is increased

A more quantitative description of the NTK at initialization is provided by its spectrum, which is shown in Fig. 12 for four different architectures. Inspecting the figure, we see that the spectrum of the NTK is heavily hierarchical, and only a few eigenvalues are actually non-zero. Such a hierarchy in the eigenvalues means that only a small subset of active directions can inform the network during training, as it will be discussed later. Note that, at least at initialization, these observations do not depend on the architecture: the eigenvalues in Fig. 12 are mostly independent of the size of the network. Even though the logarithmic scale on the vertical axis may hide some small variations, it is clear that most eigenvalues remain constant within the error bars. On the other hand, the logarithmic scale emphasizes that there are several orders of magnitude between eigenvalues for a given architecture; that hierarchical structure does not depend on the architecture.

#### NTK during training

The behavior of the NTK during training is best studied through *closure tests*, which we introduced in the previous sections. The results of the training are then compared to the known input PDFs; the performances of the training algorithm and the NN architecture are assessed by quantifying the comparison between trained PDFs and input PDFs. Following the original presentation in Ref. [24], we distinguish three levels of closure tests, which are defined by the complexity of the data used to train the NNs. We use the standard NNPDF nomenclature and refer to these three levels as level-0 (L0), level-1 (L1), and level-2 (L2) closure tests, and we denote the input PDFs used to generate the data as $f_0$. We refer the reader to the original publication for the details of the data generation at each level.

For each of the closure-test data given above, we perform a fit of the triplet combination $T_3$ using the simplified version of the NNPDF methodology that we discussed above. We initialize an ensemble of $N_{{\textrm{rep}}}= 100$ replicas with identical architecture, training each replica independently using GD optimization. Throughout the training process, we track the evolution of the NTK to understand how the network’s effective dynamics changes as it learns the target function.

***Onset of lazy training***

**Fig. 13:**
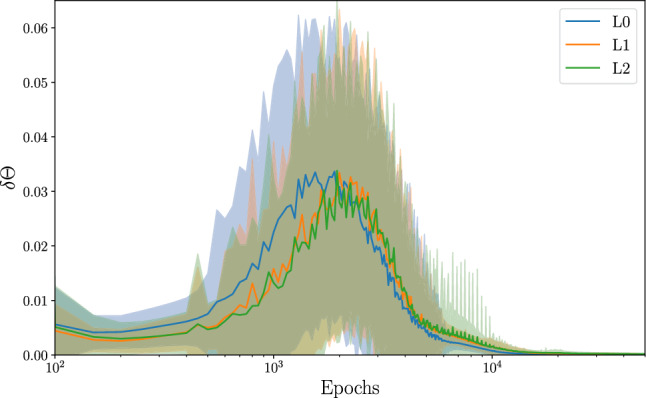
Relative variation of the NTK during training for L0, L1, and L2 data. Error bands correspond to one-sigma uncertainties over the ensemble of networks

As a first estimator of the variation of the NTK, we show in Fig. 13 the Frobenius norm of the variation during training, normalized by the Frobenius norm of the NTK itself

192 $$\begin{aligned} \delta \Theta _t = \frac{\Vert \Theta _{t+1} - \Theta _t \Vert }{\Vert \Theta _t \Vert }, \end{aligned}$$

for the three different datasets, L0, L1, and L2. Inspecting the plot reveals that the NTK undergoes significant changes during the initial phase of training, with the relative variation $\delta \Theta _t$ reaching values as high as 6%. This indicates that our settings differ from the standard picture of lazy training in the context of very wide networks, as discussed, e.g., in Refs. [20, 23, 25], where the NTK is expected to be independent of the flow time *t*. Remarkably, we do not observe a dependence on how data have been generated, indicating that the NTK dynamics is basically unaffected by the noise in the data.

After this initial phase—corresponding approximately to the first 20,000 epochs in our experiment—the NTK tends to stabilize. These two regions will be referred to as the *rich* and *lazy* training regimes, respectively, in keeping with the standard terminology adopted in the literature (see, e.g., Ref. [26] where two similar regimes were also identified). The lazy regime is discussed in detail in Sect. 7.1.1.

***Eigenvalues during training***

**Fig. 14:**
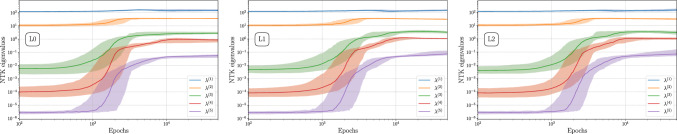
Evolution during training of the first five eigenvalues of the NTK using L0 (left), L1 (center), and L2 (right) data. Solid lines represent the median over the ensemble of networks, while solid bands correspond to 68% confidence level. Note that the subdominant eigenvalues $\lambda ^{(3)}$, $\lambda ^{(4)}$, and $\lambda ^{(5)}$ have increased by one or two orders of magnitude by the end of the rich training phase

Further insight on the evolution of the NTK can be obtained by studying its eigensystem as a function of the training time. In Fig. 14, we report the variation of the first five eigenvalues of the NTK, using the standard NNPDF architecture, for L0, L1, and L2 data. We see that the hierarchical structure observed at initialization is preserved, but the size of the subdominant eigenvalues increases significantly in the early stages of training—by one or two orders of magnitude depending on the specific eigenvalue.

**Fig. 15:**
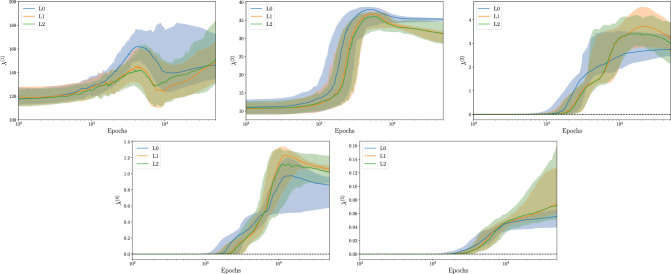
The first five eigenvalues of the NTK for L0, L1, and L2 data. Solid lines represent the median over the ensemble of networks, while solid bands correspond to 68% confidence level. Each plot corresponds to a different eigenvalue, as indicated by the label on the vertical axis. Note the different scales on the vertical axes, which reflects the hierarchy of eigenvalues discussed above. Different colors correspond to different synthetic data, the agreement between these bands confirms that the evolution of the eigensystem of the NTK does not depend on the level of noise in the data

In Fig. 15, the same first five eigenvalues of the NTK are displayed for L0, L1, and L2 data. We observe a common pattern across all data types, consistently with the observation made before in Fig. 13. This indicates the NTK evolution is insensitive to the noise included in the synthetic data. The increase of the subdominant eigenvalues, combined with the analysis of Eqs. (201) and (202) in Sect. 7 suggests that more “physical” features become learnable before lazy training sets in.

***Connection with the loss function***

**Fig. 16:**
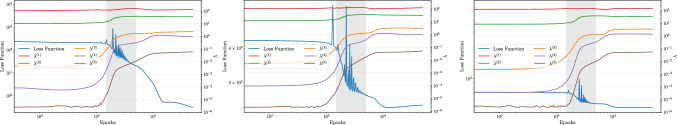
Variation of the loss function overlaid with the first five eigenvalues for a selected replica over the ensemble using L0 (left), L1 (center), and L2 (right) data. Left scale refers to the loss, while the right scale refers to the eigenvalues.

Finally, in Fig. 16, we show the variation of the loss function during training, overlaid with the first five eigenvalues of the NTK, for a selected replica over the ensemble. It is interesting to see that in correspondence with the sudden variation of the subdominant eigenvalues, the loss function drops significantly, at the cost of an instability localized in the descent. The NN is learning new features, changing its internal representation to accommodate the new information. After this initial phase, the eigenvalues stabilize and the loss function decreases smoothly, as expected in the lazy training regime. The eigenvectors corresponding to the larger eigenvalues can be interpreted as *learnable* features, while the small (or zero) eigenvalues correspond to directions in which the field *f* never evolves during training.

#### Eigenvectors as features

**Fig. 17:**
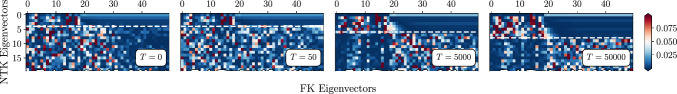
Matrix *A* as defined in Eq. (193) for L2 data and for a single replica of the NTK. The matrix is shown at different epochs of the training process, indicated in the top of each panel

It has been argued above that there is a non-trivial interplay between the eigenspace of the NTK and that of the matrix *M*. Indeed, the former encodes the model dependence, while the latter yields physical information. Of course, the two matrices are independent at initialization, and we do not expect any alignment pattern between the two. However, this picture does change during training, as the NTK evolves and the model learns the target function. To quantify this alignment, we define the matrix *A*

193 $$\begin{aligned} A_{kk'} = \left( z^{(k)}, v^{(k')} \right) ^2 = \cos ^2(\theta _{kk'}), \end{aligned}$$

where $$z^{(k)}$$ and $$v^{(k')}$$ are the *k*th and $k^{\prime }$th eigenvectors of the NTK and *M*, respectively. The matrix *A* is thus a measure of the alignment between the eigenspaces of the two matrices. The rows of the matrix correspond to the eigenvectors of the NTK, ordered by the value of the corresponding eigenvalues, with the eigenvectors corresponding to the larger eigenvalues at the top of the matrix. The columns correspond to eigenvectors of the matrix *M*, also ordered by the values of the corresponding eigenvalues, with the largest eigenvalues to the left in this case. In Fig. 17, we show the matrix *A* at different epochs of the training for L2 data and a single NTK replica.

The blue rectangle in the top-right corner of the matrix shows that the eigenvectors of the NTK corresponding to the largest eigenvalues are orthogonal to the eigenvectors of *M* that are in the kernel of *M*, i.e., to the directions that do not contribute to the observables. It is useful to remember that the largest eigenvalues of the NTK correspond to the directions that are orthogonal to $\ker \Theta$, i.e., the directions that are learnable during the training process. In order to have a robust training process, we expect these learnable directions to align with the directions that actually contribute to the loss function, which are the ones corresponding to the largest eigenvalues of *M*. Consistently with this intuition, we see that the size of this blue rectangle increases with training time. In particular, it is clear from our plot that it becomes deeper by the onset of the lazy training regime: more of the learnable directions—the *features* that the network can learn—are aligned with the directions that contribute most to the observables.

**Fig. 18:**
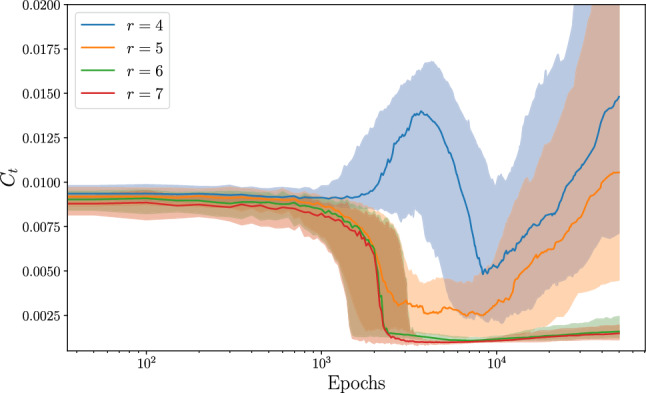
Reconstruction error $C_t(r)$ as defined in Eq. (194) as a function of the training time and for different numbers of eigenvectors *r*. Note that $f_0$ lies entirely in the subspace spanned by the first four eigenvectors of the NTK by the onset of the lazy training regime. We see that the NTK has aligned its features with the physically relevant directions of the problem

The eigenvectors of the NTK form an orthonormal basis in $\mathbb {R}^{N_{\textrm{grid}}}$ at any epoch of the training process. However, only a subset of these eigenvectors contributes to the training dynamics. The *expressivity* of the vectors belonging to the subspace orthogonal to the kernel is quantified by measuring how well the internal representation of the neural network can reconstruct the input function $f_0$ used to generate the data. We define a new figure of merit

194 $$\begin{aligned} C_t(r) = 1 - \sum _{k=1}^{r} \frac{(z^{(k)}, f_0)^2}{\Vert f_0 \Vert ^2}, \end{aligned}$$

which measures the reconstruction error of the input function $f_0$ when projected onto the subspace spanned by the first *r* eigenvectors of the NTK at training time *t*. We show $C_t(r)$ as a function of the training time and for different choices of *r* in Fig. 18. Inspecting the figure, we see that in the early stages of training the reconstruction error does not change significantly with time. This behavior is shared across all values of *r*. In fact, in this first phase, the NTK has not yet found a suitable internal representation and the inclusion of more eigenvectors corresponding to yet undiscovered directions—those associated with a small eigenvalue as in Fig. 14—does not result in an improvement of the reconstruction of $f_0$. Conversely, once the onset of lazy training is approached, we identify two distinct behaviors depending on the number of eigenvectors. If we include the eigenvectors up to the modes discovered during training (e.g., r=6 and r=7 in the figure), the reconstruction error drops significantly and remains almost constant throughout the rest of the learning process. On the other hand, if we consider fewer eigenvectors (e.g., r=4 and r=5 in the figure), the reconstruction error becomes larger and grows indefinitely with training time. The subset of eigenvectors orthogonal to $\ker \Theta$ is capable of providing an effective lower dimensional basis, provided that the new modes discovered by the NTK during training are included. Note that the reconstruction of the input function improves as long as the eigenvectors do not belong to $\ker \Theta$—the eigenvectors that belong to the kernel only add noise to the reconstruction, without bringing any new physical information.

**Fig. 19:**
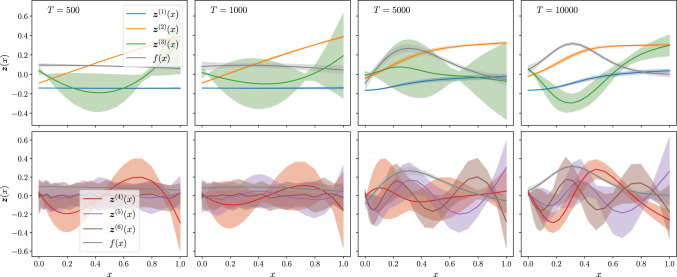
First five eigenvectors of the NTK at different training times and as function of the input *x*-grid. We also show the output of the network at the same training time, which is displayed in gray. L1 data are used

A complementary picture is displayed in Fig. 19. Here, we show the eigenvectors of the NTK at different training times as functions of the *x*-grid, denoted by $z^{(i)}$. Together with the eigenvectors, we also show the output of the trained neural network at the corresponding training time. From these plots, we see that as the training progresses, the shape of the eigenvectors becomes more structured in order to reproduce the output function. Again, this conclusion supports the observations made previously on various occasions, that during training the neural network is changing its internal representation and the NTK encodes this information.

## Lazy training regime

In the previous section, we presented an empirical study of the training dynamics through the lens of the NTK. We observed that the NTK is able to capture the main features of the training process, and that its time evolution is characterized by a rapid initial transient, followed by a slower evolution during the rest of the training. We now turn our attention on this last stage of the training, where the NTK has stabilized and becomes approximately constant. In doing so, we build upon the results presented in Refs. [23, 25] and derive the analytical solution of the flow equation, which allows us to write an explicit expression for the trained field as a function of the field at initialization and the data.

### Analytical results

#### Solution of the flow equation

The lazy training regime is characterized by a slow-evolving NTK. We denote as $t_{\textrm{ref}}$ the time at which the onset of this regime occurs. The NTK is then *frozen* to its value at $t_\textrm{ref}$, and from this time onward, the NTK is taken to be constant

195 $$\begin{aligned} \Theta _t = \Theta _{t_{\textrm{ref}}} \equiv \Theta , \quad \text {for } t \ge t_{\textrm{ref}}. \end{aligned}$$

The flow equation can then be written as

196 $$\begin{aligned} \frac{\textrm{d}}{\textrm{d}t}f_t = -\Theta M f_t + b, \end{aligned}$$

where *M* and *b* are defined as in Eq. (191). Note that now neither Θ nor *b* depend on the training time *t*. In order to solve this first-order linear differential equation, we observe that the eigenvectors of Θ

197 $$\begin{aligned} \Theta z^{(k)} = \lambda ^{(k)} z^{(k)}\, \end{aligned}$$

provide a basis for expanding Eq. (196). Furthermore, owing to the spectrum hierarchy of the NTK (Fig. 14), it is necessary to distinguish the components of $f_t$ that are in the kernel of Θ from the ones that are in the orthogonal complement. We introduce the notation

198 $$\begin{aligned}&f^\parallel _{t,k} = \left( z^{(k)}, f_t\right) , \quad \text {if}\ \lambda ^{(k)} = 0, \end{aligned}$$

199 $$\begin{aligned}&f^\perp _{t,k} = \frac{1}{\sqrt{\lambda ^{(k)}}} \left( z^{(k)}, f_t\right) , \quad \text {if}\ \lambda ^{(k)} \ne 0, \end{aligned}$$

where the scalar product has been defined as

200 $$\begin{aligned} \left( f'_{t'}, f_t\right) = \sum _{i,\alpha } f'_{t',i\alpha } f_{t,i\alpha }. \end{aligned}$$

One can readily see that the components in the kernel of Θ, $\text {ker}\ \Theta$, do not evolve during training,^9^

201 $$\begin{aligned} \frac{\textrm{d}}{\textrm{d}t}f^\parallel _{t,k} = 0 \quad \Longrightarrow \quad f^\parallel _{t,k} = f^\parallel _{0,k}. \end{aligned}$$

This means that the final solution is affected by an irreducible noise that is purely dictated by the initial condition.

The flow equation for the orthogonal components can be written as

202 $$\begin{aligned} \frac{\textrm{d}}{\textrm{d}t}f^\perp _{t,k} = - H^\perp _{kk'} f^\perp _{t,k'} + B^\perp _{k}, \end{aligned}$$

where we introduced

203 $$\begin{aligned} H^\perp _{kk'}&= \sqrt{\lambda ^{(k)}} \left( z^{(k)}, M z^{(k')}\right) \sqrt{\lambda ^{(k')}}, \end{aligned}$$

204 $$\begin{aligned} B^\perp _k&= -\sqrt{\lambda ^{(k)}} \left[ \left( z^{(k)}, M z^{(k')}\right) f^\parallel _{0,k'} - \left( z^{(k)}, (\textrm{FK})^TC_Y^{-1} Y\right) \right] . \end{aligned}$$

As discussed above, the indices on quantities that have a ⊥ suffix only span the space orthogonal to the kernel of Θ, while the indices on quantities that have a ‖ suffix span the kernel. We refer to $H^\perp$ as the flow (or training) Hamiltonian, emphasizing that training can only take place in the space orthogonal to the kernel of Θ; we see explicitly in the definition above that the flow dynamics is determined by a combination of the architecture of the NN, encoded in the NTK, and the data, on which *M* depends. More specifically, the matrix elements of *M* can be written as

205 $$\begin{aligned} \left( z^{(k)}, M z^{(k')}\right) = T^{(k)T} C_Y^{-1} T^{(k')}, \end{aligned}$$

where $T^{(k)} = T[z^{(k)}]$ is the vector of theory predictions for the data obtained using $z^{(k)}$ as the input PDF. Similarly, we have

206 $$\begin{aligned} \left( z^{(k)}, (\textrm{FK})^TC_Y^{-1} Y\right) = T^{(k)T} C_Y^{-1} Y. \end{aligned}$$

Denoting by $d^\perp$ the dimension of the subspace orthogonal to $\text {ker}\ \Theta$, $H^\perp$ is a $d^\perp \times d^\perp$ symmetric matrix, whose eigenvalues and eigenvectors satisfy

207 $$\begin{aligned} H^\perp _{kk'} w^{(i)}_{k'} = h^{(i)} w^{(i)}_{k}. \end{aligned}$$

**Fig. 20:**
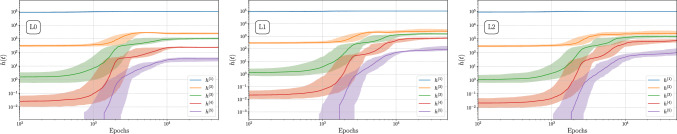
Evolution during training of the first five eigenvalues of $H^{\perp }$ using L0 (left), L1 (center), and L2 (right) data. Solid lines represent the median over the ensemble of networks, while solid bands correspond to 68% confidence level. Note that the subdominant eigenvalues $\lambda ^{(3)}$, $\lambda ^{(4)}$ and $\lambda ^{(5)}$ have increased by one or two orders of magnitude by the end of the rich training phase

**Fig. 21:**
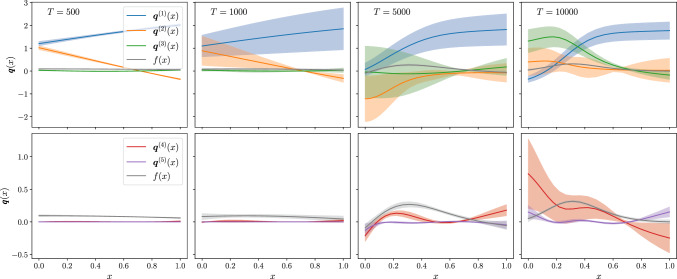
First five eigenvectors of the combined matrix H=ΘM, as in Eq. (190), at different training times and as a function of the input *x*-grid. We also show the output of the network at the same training time, which is displayed in gray. L1 data are used

In Fig. 20, we show the evolution during training of the first five eigenvalues of $H^{\perp }$ for the three different closure datasets. In Fig. 21, we show the first five eigenvectors of *H*, denoted as $q^{(i)}$, at different training times as functions of the *x*-grid. It should not come as too much of a surprise that the eigenvalues and eigenvectors of $H^{\perp }$ have a similar behavior to those of the NTK (see Figs. 14, 19), from which they are constructed. However, we see that $h^{(i)}$ are larger by around three orders of magnitude than the NTK eigenvalues.

The solution to Eq. (202) can be written as the sum of the solution of the homogeneous equation, $\hat{f}^{\perp }_{t,k}$, and a particular solution of the full equation. The solution of the homogeneous equation is

208 $$\begin{aligned} \hat{f}^{\perp }_{t,k} = \sum _{i=1}^{d^\perp } f^{\perp (i)}_{0} e^{-h^{(i)}t} w^{(i)}_k, \end{aligned}$$

where

209 $$\begin{aligned} f^{\perp (i)}_{0} = \sum _{k=1}^{d_\perp } w^{(i)}_k f^\perp _{0,k}, \end{aligned}$$

guarantees that the initial condition $\hat{f}^\perp _{t,k}=f^\perp _{0,k}$ is satisfied. Similarly, if we define

210 $$\begin{aligned} \Upsilon ^{(i)} = \sum _{k=1}^{d_\perp } w^{(i)}_k B^\perp _{k}, \end{aligned}$$

then

211 $$\begin{aligned} \check{f}^\perp _{t,k} = \mathop {{\mathop {\sum }\nolimits '}}\limits _{i} \frac{1}{h^{(i)}} \Upsilon ^{(i)} \left( 1 - e^{-h^{(i)}t}\right) w^{(i)}_k, \end{aligned}$$

where the sum only involves the non-zero modes of $H^\perp$, and is a particular solution of the inhomogeneous equation, which satisfies the boundary condition $\check{f}^{\perp }_{0,k}=0$. Hence, the solution of the flow equation in the subspace orthogonal to $\text {ker}\ \Theta$ is

212 $$\begin{aligned} f^\perp _{t,k}&= \hat{f}^\perp _{t,k} + \check{f}^\perp _{t,k} . \end{aligned}$$

Finally, collecting the parallel contribution, Eq. (201), and the solution of the orthogonal component, Eq. (212) yields a simple expression

213 $$\begin{aligned} \boxed {f_{t,\alpha } = U(t)_{\alpha \alpha '} f_{0,\alpha '} + V(t)_{\alpha I} Y_{I}. } \end{aligned}$$

The two evolution operators *U*(*t*) and *V*(*t*) have lengthy, yet explicit, expressions, which we summarize here

214 $$\begin{aligned} U(t)_{\alpha \alpha '} = \hat{U}^\perp (t)_{\alpha \alpha '} + \check{U}^\perp (t)_{\alpha \alpha '} + U^\parallel _{\alpha \alpha '}, \end{aligned}$$

where

215 $$\begin{aligned} \hat{U}^\perp (t)_{\alpha \alpha '} = \sum _{k,k'\in \perp } \sqrt{\lambda ^{(k)}} z^{(k)}_\alpha \left[ \sum _i w^{(i)}_{k} e^{-h^{(i)}t} w^{(i)}_{k'}\right] z^{(k')}_{\alpha '} \frac{1}{\sqrt{\lambda ^{(k')}}}, \end{aligned}$$

and

216 $$\begin{aligned} U^\parallel _{\alpha \alpha '} = \sum _{k''\in \parallel } z^{(k)}_\alpha z^{(k)}_{\alpha '}. \end{aligned}$$

The contributions from $\check{U}^\perp (t)$ and *V*(*t*) are more easily expressed by introducing the operator

217 $$\begin{aligned} \mathcal {M}(t)_{\alpha \alpha '} = \sum _{k,k'\in \perp } \sqrt{\lambda ^{(k)}} z^{(k)}_\alpha \left[ \mathop {{\mathop {\sum }\nolimits '}}\limits _{i} w^{(i)}_{k} \frac{1}{h^{(i)}}\, \left( 1- e^{-h^{(i)}t}\right) w^{(i)}_{k'}\right] z^{(k')}_{\alpha '} \sqrt{\lambda ^{(k')}}. \end{aligned}$$

Then, we can write

218 $$\begin{aligned} \check{U}^\perp (t) = - \mathcal {M}(t)\; (\textrm{FK})^TC_Y^{-1} (\textrm{FK})\left[ \sum _{k''\in \parallel } z^{(k'')} z^{(k'') T}\right] , \end{aligned}$$

and

219 $$\begin{aligned} V(t) = \mathcal {M}(t)\; (\textrm{FK})^TC_Y^{-1}, \end{aligned}$$

where we note that the term in the bracket in Eq. (218) is simply the projector on the kernel of the NTK. The four terms that appear in the analytical solution have a clear physical interpretation:

- The first term $\hat{U}^\perp (t)$ suppresses the components of the initial condition that lie in the subspace orthogonal to the kernel of the NTK. These are the components that are learned by the network during training. While the trained solution still depends on its value at initialization, that dependence is suppressed during training. This suppression is exponential in the training time, and the rates are given by the eigenvalues of $H^{\perp }$.
- The contribution from $U^\parallel$ yields the component of the initial condition that lies in the kernel of the NTK. As such, those components remain unchanged during training and are part of the trained field at all times *t*.
- The two remaining contributions are best understood by combining them together
  220 $$\begin{aligned} \check{U}^{\perp }(t) f_{0} + V(t) Y = \mathcal {M}(t)\; (\textrm{FK})^TC_Y^{-1} \left[ Y - (\textrm{FK})f_{0}^{\parallel }\right] . \end{aligned}$$
  The parallel component of the initial condition $f_{0}^{\parallel }$ does not evolve during training, and therefore, it yields a contribution $(\textrm{FK})f_{0}^{\parallel }$ to the theoretical prediction of the data points at all times *t*. This is taken into account by subtracting this contribution from the data, before the inference is performed.

##### Exercise 9

Derive the analytic solution in Eq. (213) by solving the flow equation in the subspace orthogonal to the kernel of the NTK, and then adding the contribution from the kernel, as explained in the text. In doing so, you will need to compute the eigenvalues and eigenvectors of $H^\perp$, and use them to write the solution of the homogeneous and inhomogeneous equations. Finally, you will need to express the solution in terms of the initial condition $f_0$ and the data *Y* by using the definitions of $B^\perp$ and $H^\perp$.

**Fig. 22:**
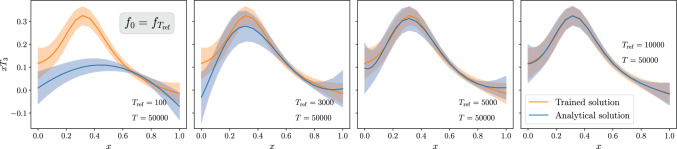
Comparison of the trained and analytical evolution at the end of training. Each panel corresponds to a different frozen NTK, whereby the analytical solution is computed starting from $f_{T_{\textrm{Ref}}}$. The orange curve represents the final trained function after 50,000 iterations of GD, and is the same across panels. Error bands represent one-sigma uncertainties across replicas. L2 data are used

The solution in Eq. (213) is the main result of this section. It shows that the training process can be described as the sum of a linear transformation of the initial fields $f_{0,\alpha }$, and a linear transformation of the data $Y_I$. The two transformations depend on the flow time *t* and are given by the evolution operators *U*(*t*) and *V*(*t*). Figure 22 compares the analytical solution with the trained function at the end of training, for different choices of the frozen NTK. The NN is trained using the numerical GD until $t_{\textrm{ref}}$, at which point the NTK is frozen. The evolution time *t* used in the analytical solution is the difference between the total training time and $t_{\textrm{ref}}$; the initial condition for the analytical solution is the trained solution at $t_{\textrm{ref}}$. Central value and uncertainty bands are obtained by computing the analytical solution for each replica of the initial condition and frozen NTK.^10^ The figures agree with the expectation; the closer $t_{\textrm{ref}}$ is to the onset of the lazy regime, the better the agreement between the analytical solution and the trained function.

**Fig. 23:**
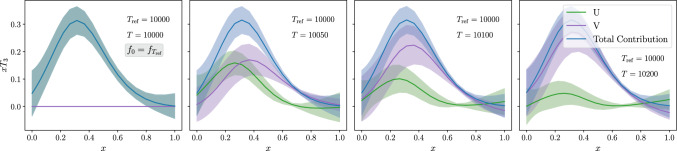
Decomposition of the analytical solution into the two contributions from *U* and *V* at different training times. The frozen NTK is fixed across panels, and corresponds to the one at $T_{\textrm{ref}} = 10{,}000$. The initial condition for the analytical solution is always $f_{T_{\textrm{ref}}}$. As in Fig. 22, L2 data are used

A complementary perspective is provided in Fig. 23, where the analytical solution is decomposed into the two contributions from *U* and *V*. In each panel, the initial condition $f_{t_{\textrm{ref}}}$ is evolved analytically for different training times by keeping the frozen NTK fixed. We see that as training proceeds, the contribution from *U* is progressively suppressed, in accordance with the observation made above. On the other hand, the contribution from *V* grows and becomes dominant at later epochs, indicating that the trained function is mostly determined by the data, rather than the initial condition of the network. We also observe that such behavior happens quite rapidly—in a training time interval $\Delta T \approx 200$—as a consequence of the fact that the time scales in the analytical solution are determined by the inverse of the eigenvalues of $H^\perp$, and the latter are typically large.

The analytical solution in Eq. (213) sheds a new light onto the behavior of the numerical training of a neural network. Given these results, it is natural to ask whether the information encoded in the NTK alone can drive training, independently of the initial condition, i.e., whether the analytical solution can be used to perform kernel learning. We address this question in Sect. 7.2, after deriving a few additional analytical results in the subsections below.

#### Crosschecks using L0 data

The analytical solution enables rigorous validation of our implementation through crosschecks with L0 data, where we have complete control over the data generation process. In this case, the realization of the dataset is completely determined by the input PDFs

221 $$\begin{aligned} Y = (\textrm{FK}){f_0}. \end{aligned}$$

Note that using L0 data only affect the second term in Eq. (213).^11^ We can then rewrite the combined term in Eq. (220) as follows:

222 $$\begin{aligned} \check{U}^\perp (t) f_0 + V(t) Y&= \mathcal {M}(t)\, (\textrm{FK})^TC_{Y}^{-1} (\textrm{FK})\, \left[ {f_0}- f_{0}^\parallel \right] . \end{aligned}$$

The subtraction taking place in the square brackets of Eq. (222) suggests that the effective function that the neural network actually sees is not the input function ${f_0}$ used to generate the data, but rather the difference between ${f_0}$ and the component of the initial function $f_0$ that lies in the subspace spanned by the kernel of the NTK, i.e., $f_0^\parallel$. In other words, the parallel component $f_0^\parallel$, which, we remind the reader, does not evolve during the analytic training, acts as a constant “bias” in the training process, effectively shifting the input function seen by the neural network. Of course, the actual magnitude of this irreducible noise depends both on how $f_0$ and the kernel of the NTK are distributed over the ensemble.

**Fig. 24:**
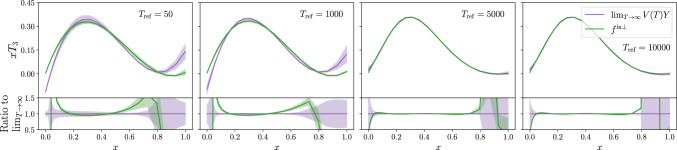
Test of the $t\rightarrow \infty$ limit of the L0 training for different frozen NTKs. The green curve represents the projection of the input function ${f_0}$ onto the subspace orthogonal to the kernel of the NTK at $t_{\textrm{ref}}$, i.e., ${f_0^{\perp }}$. The purple curve represents the contribution of the operator *V*, computed with the NTK at $T_{\textrm{ref}}$, in the limit of infinite training time

Note that the observation above remains true even in the limit of infinite training

223 $$\begin{aligned} \lim _{t\rightarrow \infty } V(t) Y = {f_0^{\perp }}+ \mathcal {M}_{\infty } M {f_0^{\parallel }}, \end{aligned}$$

which shows that the *V* component of the trained solution reproduces exactly the component of the PDF that lies in the subspace orthogonal to the kernel of Θ. We compare the asymptotic behavior of *V*(*t*)*Y* and ${f_0^{\perp }}$ in Fig. 24, where we see that the analytical solution at infinite training time reproduces the expected result, i.e., it coincides with ${f_0^{\perp }}$, as long as $T_{\textrm{ref}}> 1000$. The second term in the square bracket on the right-hand side of Eq. (222) is the contribution from the parallel component at initialization that does not evolve in the training process. Given that $f_0$ is almost normally distributed around zero, that term does not contribute to the central value of the fitted PDF, i.e., to the average of the trained solution over replicas.

**Fig. 25:**
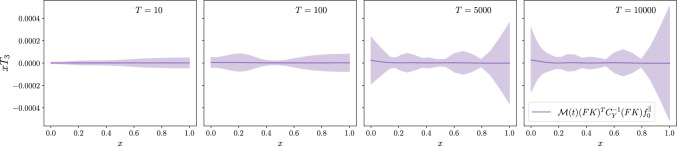
Test of the average of the parallel contribution for different epochs. The reference epoch at which the frozen NTK is chosen is $T_{\textrm{ref}} = 10{,}000$. L2 data are used in the plot. Note that the scale on the vertical axis is three orders of magnitude smaller than in Fig. 24

The time evolution of

224 $$\begin{aligned} \mathbb {E}\left[ \mathcal {M}(t)\, (\textrm{FK})^TC_{Y}^{-1} (\textrm{FK})\, f_{0}^\parallel \right] \, \end{aligned}$$

is shown in Fig. 25.

#### Infinite training time

In the limit of infinite training time, the evolution operators *U*(*t*) and *V*(*t*) simplify and yield an elegant interpretation of the minimum of the cost function. For large training times, we have

225 $$\begin{aligned} \hat{U}^\perp _{\infty , \alpha \alpha '}&= \lim _{t\rightarrow \infty }\hat{U}^\perp (t)_{\alpha \alpha '} = 0\, \end{aligned}$$

226 $$\begin{aligned} \mathcal {M}_{\infty , \alpha \alpha '}&= \lim _{t\rightarrow \infty }\mathcal {M}(t)_{\alpha \alpha '} = \sum _{k,k'\in \perp } \sqrt{\lambda ^{(k)}} z^{(k)}_\alpha \left[ \mathop {{\mathop {\sum }\nolimits '}}\limits _{i} w^{(i)}_{k} \frac{1}{h^{(i)}}\, w^{(i)}_{k'}\right] z^{(k')}_{\alpha '} \sqrt{\lambda ^{(k')}}, \end{aligned}$$

and explicit expressions for $\check{U}^\perp _{\infty }$ and $V_{\infty }$ are obtained from $\mathcal {M}_{\infty }$. The term in the square bracket in Eq. (226) is the spectral decomposition of the pseudo-inverse of $H^\perp$ in $d_\perp$-dimensional orthogonal subspace. Therefore, the operator $\mathcal {M}_{\infty }$ acts as follows on a field $f_{\alpha }$:

1. The term on the right of the square bracket computes the coordinate $f_{k'}$ introduced in Eq. (199). The $f_k$ are the coordinates of the component $f^\perp$ that evolves during training
  227 $$\begin{aligned} f^\perp = \sum _{k\in \perp } \sqrt{\lambda ^{(k)}} f_k\, z^{(k)}. \end{aligned}$$
2. The term in the square bracket applies the pseudo-inverse to the coordinates $f_k$
  228 $$\begin{aligned} f'_k = \left( H^\perp \right) ^+_{kk'} f_{k'}. \end{aligned}$$
3. The final term on the left of the square bracket reconstructs the full field corresponding to the coordinates $f'_{k}$
  229 $$\begin{aligned} f^{'\perp } = \sum _{k\in \perp } \sqrt{\lambda ^{(k)}} f'_{k}\, z^{(k)}. \end{aligned}$$

As discussed at the end of Sect. 7.1.1 it is convenient to combine the contributions of $\check{U}^\perp _{\infty }$ and $V_{\infty }$

230 $$\begin{aligned} \check{U}^{\perp }_{\infty } f_{0} + V_{\infty } Y = \mathcal {M}_{\infty }\; (\textrm{FK})^TC_Y^{-1} \left[ Y - (\textrm{FK})f_{0}^{\parallel }\right] . \end{aligned}$$

The contribution to the observables from the parallel components of *f* does not change during training; therefore, that contribution is subtracted from the data and the orthogonal components of *f* are adjusted to minimize the $\chi ^2$ of the corrected data. The minimum of the $\chi ^2$ in the orthogonal subspace is found applying $\mathcal {M}_{\infty }$, i.e., by projecting in the orthogonal subspace, applying the pseudo-inverse and finally recomputing the full field as detailed above.

### Numerical results

The results shown in Sects. 7.1.1 and 6.2.2 support the idea that the NTK is capable to encode in its eigenvectors the physical features learned during training. Let us now study the analytical solution that we obtain by choosing the initial condition to be an ensemble of networks at initialization as in Fig. 10. The analytical solution is computed using the NTK frozen at $t_{\textrm{ref}}$.

#### Central value and covariance of the trained fields

The analytical solution in Eq. (213) is inherently stochastic, since the frozen NTK at $t_{\textrm{ref}}$ is actually obtained from an ensemble of networks. The central value of the analytical solution is defined as

231 $$\begin{aligned} \bar{f}_{t,\alpha } = \mathbb {E}\left[ f_{t,\alpha }\right] = \mathbb {E}\left[ U(t)_{\alpha \alpha '} f_{0,\alpha '}\right] + \mathbb {E}\left[ V(t)_{\alpha I} Y_I\right] . \end{aligned}$$

More interestingly, the covariance matrix at any time *t* is given by

232 $$\begin{aligned} \textrm{Cov}[f_t,f_t^T]&= \mathbb {E}\left[ U(t) f_0 f_0^T U(t)^T\right] - \mathbb {E}\left[ U(t) f_0\right] \mathbb {E}\left[ f_0^T U(t)^T\right] \nonumber \\&\quad + \mathbb {E}\left[ U(t) f_0 Y^T V(t)^T\right] - \mathbb {E}\left[ U(t) f_0\right] \mathbb {E}\left[ Y^T V(t)^T\right] \nonumber \\&\quad + \mathbb {E}\left[ V(t) Y f_0^T U(t)^T\right] - \mathbb {E}\left[ V(t) Y\right] \mathbb {E}\left[ f_0^T U(t)^T\right] \nonumber \\&\quad + \mathbb {E}\left[ V(t) Y Y^T V(t)^T\right] - \mathbb {E}\left[ V(t) Y\right] \mathbb {E}\left[ Y^T V(t)^T\right] . \end{aligned}$$

Note that the first and the fourth lines above yield symmetric matrices, while the third line is just the transpose of the second, thereby ensuring that the whole covariance matrix is the sum of three symmetric matrices and therefore is symmetric

233 $$\begin{aligned} \textrm{Cov}[f_t,f_t^T] = C_t^{(00)} + C_t^{(0Y)} + C_t^{(YY)}, \end{aligned}$$

where

234 $$\begin{aligned} C_t^{(00)}&= \mathbb {E}\left[ U(t) f_0 f_0^T U(t)^T\right] - \mathbb {E}\left[ U(t) f_0\right] \mathbb {E}\left[ f_0^T U(t)^T\right] , \end{aligned}$$

235 $$\begin{aligned} C_t^{(0Y)}&= \mathbb {E}\left[ U(t) f_0 Y^T V(t)^T\right] - \mathbb {E}\left[ U(t) f_0\right] \mathbb {E}\left[ Y^T V(t)^T\right] \nonumber \\&\quad + \mathbb {E}\left[ V(t) Y f_0^T U(t)^T\right] - \mathbb {E}\left[ V(t) Y\right] \mathbb {E}\left[ f_0^T U(t)^T\right] , \end{aligned}$$

236 $$\begin{aligned} C_t^{(YY)}&= \mathbb {E}\left[ V(t) Y Y^T V(t)^T\right] - \mathbb {E}\left[ V(t) Y\right] \mathbb {E}\left[ Y^T V(t)^T\right] . \end{aligned}$$

Equation (233) shows explicitly the various contributions to the covariance matrix. Indeed, $C_t^{(00)}$ quantifies the contribution to the covariance matrix that is purely due to the fluctuations of the initial condition, while $C_t^{(YY)}$ quantifies the contribution that is purely due to the statistical fluctuations of the data. The mixed term $C_t^{(0Y)}$ accounts for the correlations between the two sources of uncertainty.

#### Convergence of the analytical solution

In order to study the convergence of the analytical solution, we compare the following:

- the analytical solution (AS), obtained using an ensemble of networks at initialization as the initial condition;
- the trained solution (TS), obtained by training another ensemble of networks, drawn from the same prior distribution, using GD.

**Fig. 26:**
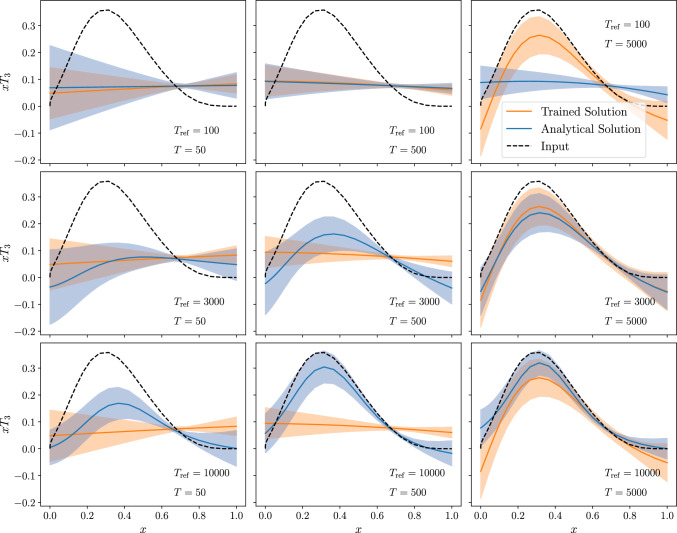
Comparison of the trained (orange) and analytical (blue) evolution starting from an ensemble of networks at initialization as the initial condition. Each row corresponds to a different frozen NTK, while the columns represent different training times. The dashed line represents the input function used to generate the synthetic data, i.e., the *true* result. L2 data are used

This comparison is shown in Fig. 26 for L2 data, where the rows in the grid correspond to different frozen NTKs, while the columns represent numerical and analytical evolution after T=50,500 and 5000 epochs. These results deserve a few comments.

The first observation is that the NTK at early stages of training is not able to drive the prior toward the true function, as shown in the first and, though less dramatically, in the second row of Fig. 26. This is expected, as we extensively discussed in Sect. 6.2.2, since, at early stages of training by GD, the NTK has not yet aligned its internal representation with the data.

More significantly, we observe a significant discrepancy between the AS and the TS even at T=5000. At the beginning of training, there is clearly no difference between AS and TS since both represent the neural network output at initialization, with variations due only to different initialization seeds. During early training stages, AS and TS differ as expected. Indeed, the analytical solution is computed using the frozen NTK at $t_{\textrm{ref}}$, while the trained solution evolves with an NTK that is still changing, as shown in Sect. 6. Crucially, if the NTK at $t_{\textrm{ref}}$ has already learned from the data and aligned with the solution, the AS converges faster to the target, while the TS requires additional epochs before evolving in the correct direction.

#### Connection with linear methods

We can consider a simplifying limit of Eq. (231), where the initial condition $f_0$ and the data *Y* are statistically independent from the respective evolution operators *U*(*t*) and *V*(*t*). Note that the first term on the right-hand side of Eq. (231) can only be non-zero because of the correlations between *U*(*t*) and $f_0$. In the absence of such correlations, the first term would be given by the product of the expectation values and, therefore, would vanish if $f_0$ is an ensemble of networks at initialization. Under these assumptions, we have

237 $$\begin{aligned} \bar{U}(t)&= \mathbb {E}\left[ U(t)\right] , \end{aligned}$$

238 $$\begin{aligned} \bar{V}(t)&= \mathbb {E}\left[ V(t)\right] , \end{aligned}$$

and

239 $$\begin{aligned} \bar{f}_{t,\alpha } = \bar{U}(t)_{\alpha \alpha '} \bar{f}_{0,\alpha '} + \bar{V}(t)_{\alpha I} Y_I = \bar{V}(t)_{\alpha I} Y_I. \end{aligned}$$

The second term in Eq. (231), or equivalently Eq. (239), explicitly shows the contribution of each data point to the central value of the trained fields at each value of $x_{\alpha }$. It is worthwhile remarking that in this limit, the central value from the set of trained networks is a linear combination of the data points, with coefficients given by the evolution operator $V(t)_{\alpha I}$.

**Fig. 27:**
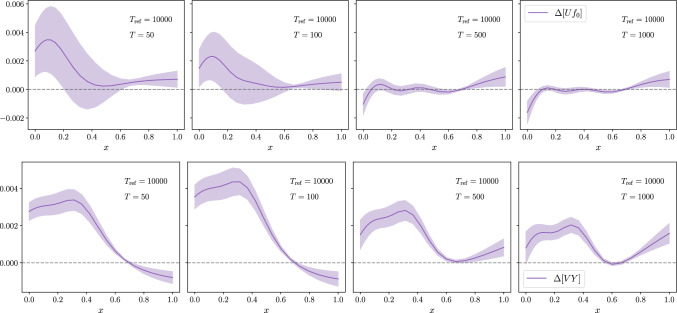
Behavior of $\Delta [U(t)f_0]$ and Δ[V(t)Y], as defined in Eqs. (240) and (241), as functions of the training time. The operators *U*(*T*) and *V*(*T*) are constructed by taking the NTK at $T_{\textrm{ref}} = 10{,}000$, which is fixed across panels. The uncertainties are extracted from the bootstrap ensemble as discussed in the text

In the absence of general theorems, we verify this assumption empirically. From the ensemble of replicas, we generate bootstrap samples and compute the following two estimators:

240 $$\begin{aligned} \Delta [U(t)f_0]&= \mathbb {E}\left[ U(t) f_{0}\right] - \mathbb {E}\left[ U(t) \right] \mathbb {E}\left[ f_{0}\right] , \end{aligned}$$

241 $$\begin{aligned} \Delta [V(t)Y]&= \mathbb {E}\left[ V(t) Y\right] - \mathbb {E}\left[ V(t) \right] \mathbb {E}\left[ Y\right] . \end{aligned}$$

for different training times, using the same frozen NTK and L2 data. The results are shown in Fig. 27 for the *U* (upper panel) and *V* (lower panel) contributions. The error bands are computed using bootstrap error. By inspecting the figures, we see two distinct patterns emerging. For the operator *U*, $\Delta [U(t) f_0]$ is different from zero for small training times, and thus, the correlations between *U*(*t*) and $f_0$ are non-negligible. However, as training proceeds, $\Delta [U(t) f_0]$ becomes compatible with zero within the error bars, suggesting that the correlations are progressively suppressed. The case of the *V* operator is even more striking, as Δ[V(t)Y] is clearly non-negligible across all training times, although it also shows a decreasing trend as training proceeds. This suggests that the correlations between *V*(*t*) and *Y* cannot be neglected.

Let us conclude this brief discussion by noting that Eq. (239) resembles the structure of a linear method, like Backus–Gilbert or Gaussian Processes.

#### Error decomposition

**Fig. 28:**
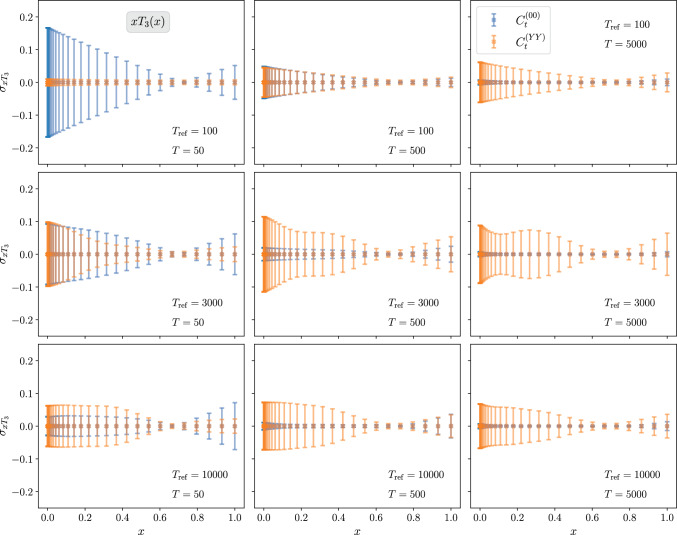
Decomposition of the error budget of the trained fields into the two components from the initial condition (blue) and from the data (orange), as defined in Eqs. (234) and (236). Each row corresponds to a different frozen NTK, while the columns represent different training epochs. L2 data are used. We see that if the NTK is taken at later stages of training, the contribution from the initial condition is severely suppressed toward the end of training

The analytical expression for the covariance matrix, Eq. (233), allows us to monitor the relative size of the three contributions as training proceeds. For a properly trained ensemble of networks, the covariance of the trained fields should be dominated by the statistical error on the data. We show the diagonal entries of the two contributions $C_t^{(00)}$ (blue band) and $C_t^{(YY)}$ (orange band) to the error budget in Fig. 28, for different frozen NTKs (rows) and different training epochs (columns), using L2 data as before. We do not show the mixed term $C_t^{(0Y)}$, since it is negligible with respect to the other two contributions—the two sources of uncertainty are largely uncorrelated. In general, we observe that toward the end of the training process, the contribution from the data $C_t^{(YY)}$ becomes dominant with respect to the contribution coming from the initial condition $C_t^{(00)}$. We also see that the suppression of the initial condition is more severe and happens earlier when the frozen NTK is taken at later stages of training.

**Fig. 29:**
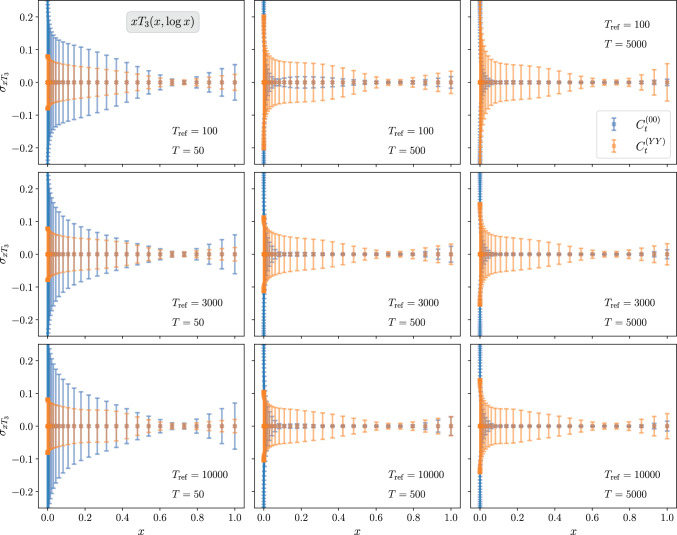
Similar to Fig. 28, but now for the case of scaled input f(x,logx)

In order to study the dependence of the error decomposition on the initial condition, we repeat the same analysis for the case of scaled input f(x,logx), i.e. with two neurons in the input layer. The initial condition $f_0$ of the analytical solution is drawn from the same prior distribution as the trained solution, and corresponds to the orange curve in Fig. 10. The resulting error decomposition is shown in Fig. 29 for L2 data, where panels are ordered as in Fig. 28. Inspecting the figures, we observe that now the contribution of the initial condition becomes dominant in the small-*x* region, even for large training times and irrespective of the epoch at which the NTK is frozen. This result reflects the behavior of the prior distribution at small-*x*, where indeed error bands are significantly enlarged with respect to the case of linear input. Interestingly, even the contribution from the data, $C^{(YY)}_t$, increases at small-*x* toward larger training times. This can be explained by observing that, despite not being explicitly dependent on the initial condition $f_0$, the evolution operator *V*(*t*) is constructed from a frozen NTK that has encoded the dependence on the architecture through the training process [see Eq. (219)]. That the difference between Figs. 28 and 29 is primarily localized at small-*x* showcases that, for the region left uncovered by the data, the methodology is not able to suppress the dependence on the initial condition. In fact, where the information from the data is available (corresponding roughly to x≳0.01), the dependence on the initial condition lessens as the analytical solution evolves (left to right). This reduction occurs more rapidly for frozen NTKs taken at later stages of training (top to bottom), showing that there is a non-trivial interplay between the information provided by the data and that acquired by the NTK.

These studies reveal the intricate connection between the prior distribution and the uncertainties of PDFs. In the region constrained by data, the error is dominated by the statistical error on the data, rather than by the fluctuations of the initial fields. This is an important step in our study of the error estimates. It guarantees that the error bars computed from the ensemble of trained PDFs are not biased by the choice of prior, which depends on the selected architecture, activation function, and probability distributions for the biases and weights at initialization.

## Attempts to a synthesis

Let us try to conclude these lectures by summarizing some lessons we learned along the way.

First of all, it is worthwhile emphasizing that the discussion of the training based on the NTK can be adapted to any parametrization and minimization process, including fixed functional forms [3, 27, 28] or kernels [29]. What information can be extracted from the NTK in those cases is an open question for future investigations, aiming at comparing different procedures within a unified framework.

From a more general perspective, we can trace the difficulties in solving inverse problems to the fact that the initial question is ill-posed. A stable solution can only be obtained by regularizing the problem in one way or another.

The probabilistic approach that we have advocated here has the advantage of casting different approaches as alternative ways to regularize the problem and define a posterior distribution for the solution. Having analyzed in detail Backus–Gilbert, Gaussian Processes, and Neural Network solutions, we have been able to expose some relations between these different approaches. In particular, we found that the BG solution can be understood as special case of the GP solution. A careful analysis of the systematic errors in these two frameworks will allow for more robust results.

It is interesting to remark that the GP solution is based on Bayesian inference, while the other two rely on minimizing some figure of merit. In particular, for the case of Neural Networks, the solution is obtained by finding an *optimal* stopping time, rather than letting the minimization reach an absolute minimum that would correspond to an overfitted solution. Understanding the connection between the stopping condition and the results obtained with BG/GP methods is an open question, which will shed light on the errors quoted with these different methods. Understanding the training dynamics of NNs is a fascinating problem, and we have only started to scratch the surface of the problem.

## Data Availability

No new data were created or analyzed in this study.

## Footnotes

1 For instance, an interesting review [1] has appeared as we were finishing writing these lectures, which covers a number of topics that we have not discussed here.

2 We use a tilde to identify the posterior distributions in these lectures. As an alternative, we also use the notation $P(f | D_{N_{\textrm{dat}}})$ to denote the posterior distribution of *f* given the data $D_{N_{\textrm{dat}}}$, where $D_{N_{\textrm{dat}}}$ is the set of $N_{\textrm{dat}}$ measurements. The two notations are equivalent, and we will use them interchangeably.

3 The reader can refer to the examples of the previous section for concrete instances of the functions $C_I(x)$ in the case of PDFs and lattice QCD.

4 We choose here to measure Euclidean time in physical units. The (dimensionless) number of points in the time direction is $N_T=T/a$.

5 We discussed at the beginning of this section the possibility of sampling the posterior distribution of the hyperparameters, which would be the most rigorous way to proceed. This has not been done in Ref. [12], where the hyperparameters were kept fixed at the mode of the posterior distribution.

6 The result shown in Fig. 4 is obtained from a pseudoscalar correlator of fermions in a higher representation, computed within the ensemble B3 generated by the authors in Ref. [15].

7 The notation here refers to the matrix element $(\alpha _1,\alpha _2)$ of the inverse matrix of $\gamma ^{(\ell )}$, and *not* to the inverse of the matrix element $\gamma ^{(\ell )}_{\alpha _1\alpha _2}$.

8 Note that, in addition to the scaling $\mathcal {O}(1/n)$ theoretically predicted for large networks, the uncertainty bands include bootstrap errors due to the finite size of the ensemble. Using an ensemble of 100 replicas, the bootstrap error on the standard deviation is $\sim 10\%$.

9 Despite this result having been obtained using the frozen NTK, it is worth mentioning that at any time during training the kernel of the NTK is always defined and in general non-empty. Hence, even in the initial stages of the training process, there is a component that is completely determined by the initial condition, i.e., by the prior distribution in functional space.

10 Unless stated otherwise, in this section, central values and uncertainties are always computed as ensemble averages across replicas.

11 To be more precise, since the analytical solution requires the NTK to be frozen at a certain epoch $T_{\textrm{ref}}$, the NTK also depends on the data used in the training.

## References

[1] A. Rothkopf, *Tackling Inverse Problems for PDFs From Lattice QCD* (2026)

[2] R.D. Ball et al., The path to proton structure at 1% accuracy. Eur. Phys. J. C **82**(5), 428 (2022). 10.1140/epjc/s10052-022-10328-7. arXiv:2109.02653 [hep-ph]

[3] S. Bailey, T. Cridge, L.A. Harland-Lang, A.D. Martin, R.S. Thorne, Parton distributions from LHC, HERA, Tevatron and fixed target data: MSHT20 PDFs. Eur. Phys. J. C **81**(4), 341 (2021). 10.1140/epjc/s10052-021-09057-0. arXiv:2012.04684 [hep-ph]

[4] T.-J. Hou et al., New CTEQ global analysis of quantum chromodynamics with high-precision data from the LHC. Phys. Rev. D **103**(1), 014013 (2021). 10.1103/PhysRevD.103.014013. arXiv:1912.10053 [hep-ph]

[5] M. Salg, F. Romero-López, W.I. Jay, Bayesian analysis and analytic continuation of scattering amplitudes from lattice QCD. Phys. Rev. D **112**(11), 114502 (2025). 10.1103/ty19-xvvw. arXiv:2506.16161 [hep-lat]

[6] R. Penrose, A generalized inverse for matrices. Math. Proc. Camb. Philos. Soc. **51**(3), 406–413 (1955). 10.1017/S0305004100030401

[7] G.E. Backus, J.F. Gilbert, Numerical applications of a formalism for geophysical inverse problems. Geophys. J. Int. **13**(1–3), 247–276 (1967). 10.1111/j.1365-246X.1967.tb02159.x

[8] A.P. Valentine, M. Sambridge, Gaussian process models—I. A framework for probabilistic continuous inverse theory. Geophys. J. Int. **220**(3), 1632–1647 (2019). 10.1093/gji/ggz520

[9] M. Hansen, A. Lupo, N. Tantalo, Extraction of spectral densities from lattice correlators. Phys. Rev. D **99**(9), 094508 (2019). 10.1103/PhysRevD.99.094508. arXiv:1903.06476 [hep-lat]

[10] A. Candido, L. Del Debbio, T. Giani, G. Petrillo, Bayesian inference with Gaussian processes for the determination of parton distribution functions. Eur. Phys. J. C **84**(7), 716 (2024). 10.1140/epjc/s10052-024-13100-1. arXiv:2404.07573 [hep-ph]

[11] A.C. Benvenuti et al., A high statistics measurement of the proton structure functions F(2) (x, Q**2) and R from deep inelastic muon scattering at high Q**2. Phys. Lett. B **223**, 485–489 (1989). 10.1016/0370-2693(89)91637-7

[12] L. Del Debbio, A. Lupo, M. Panero, N. Tantalo, Bayesian solution to the inverse problem and its relation to Backus-Gilbert methods. Eur. Phys. J. C **85**(2), 185 (2025). 10.1140/epjc/s10052-025-13885-9. arXiv:2409.04413 [hep-lat]

[13] J. Horak, J.M. Pawlowski, J. Rodríguez-Quintero, J. Turnwald, J.M. Urban, N. Wink, S. Zafeiropoulos, Reconstructing QCD spectral functions with Gaussian processes. Phys. Rev. D **105**(3), 036014 (2022). 10.1103/PhysRevD.105.036014. arXiv:2107.13464 [hep-ph]

[14] J. Bulava, M.T. Hansen, M.W. Hansen, A. Patella, N. Tantalo, Inclusive rates from smeared spectral densities in the two-dimensional O(3) non-linear -model. JHEP **07**, 034 (2022). 10.1007/JHEP07(2022)034. arXiv:2111.12774 [hep-lat]

[15] L. Del Debbio, A. Lupo, M. Panero, N. Tantalo, Multi-representation dynamics of SU(4) composite Higgs models: chiral limit and spectral reconstructions. Eur. Phys. J. C **83**(3), 220 (2023). 10.1140/epjc/s10052-023-11363-8. arXiv:2211.09581 [hep-lat]

[16] C. Alexandrou et al., Probing the energy-smeared R ratio using lattice QCD. Phys. Rev. Lett. **130**(24), 241901 (2023). 10.1103/PhysRevLett.130.241901. arXiv:2212.08467 [hep-lat] 37390427 10.1103/PhysRevLett.130.241901

[17] E. Bennett, *Meson Spectroscopy From Spectral Densities in Lattice Gauge Theories* (2024). arXiv:2405.01388 hep-lat

[18] G. Backus, F. Gilbert, The resolving power of gross earth data. Geophys. J. Int. **16**(2), 169–205 (1968). 10.1111/j.1365-246X.1968.tb00216.x

[19] X. Glorot, Y. Bengio, Understanding the difficulty of training deep feedforward neural networks, in *Proceedings of the Thirteenth International Conference on Artificial Intelligence and Statistics. Proceedings of Machine Learning Research*, vol. 9, ed. by Y.W. Teh, M. Titterington (PMLR, Chia Laguna Resort, Sardinia, Italy, 2010), p. 249–256. https://proceedings.mlr.press/v9/glorot10a.html

[20] D.A. Roberts, S. Yaida, B. Hanin, *The Principles of Deep Learning Theory: An Effective Theory Approach to Understanding Neural Networks* (Cambridge University Press, Cambridge, 2022). 10.1017/9781009023405

[21] A. Chiefa, L. Del Debbio, R. Kenway, *Quantitative Understanding of PDF Fits and Their Uncertainties* (2025) arXiv:2512.24116 [hep-ph] 10.1140/epjc/s10052-026-16008-0 PMC13310837 42375711

[22] D.G.T. Barrett, B. Dherin, *Implicit Gradient Regularization* (2022). arXiv:2009.11162 [cs.LG]

[23] A. Jacot, F. Gabriel, C. Hongler, Neural tangent kernel: convergence and generalization in neural networks. Adv. Neural. Inf. Process. Syst. **31** (2018). arXiv:1806.07572 cs.LG

[24] R.D. Ball et al., Parton distributions for the LHC Run II. JHEP **04**, 040 (2015). 10.1007/JHEP04(2015)040. arXiv:1410.8849 [hep-ph]

[25] J. Lee, L. Xiao, S. Schoenholz, Y. Bahri, R. Novak, J. Sohl-Dickstein, J. Pennington, Wide neural networks of any depth evolve as linear models under gradient descent. J. Stat. Mech: Theory Exp. **2020**(12), 124002 (2020). 10.1088/1742-5468/abc62b. arXiv:1902.06720 stat.ML

[26] S. Fort, G.K. Dziugaite, M. Paul, S. Kharaghani, D.M. Roy, S. Ganguli, Deep learning versus kernel learning: an empirical study of loss landscape geometry and the time evolution of the neural tangent kernel. Adv. Neural. Inf. Process. Syst. **33**, 5850–5861 (2020). arXiv:2010.15110 cs.LG

[27] A. Ablat et al., New results in the CTEQ-TEA global analysis of parton distributions in the nucleon. Eur. Phys. J. Plus **139**(11), 1063 (2024). 10.1140/epjp/s13360-024-05865-x. arXiv:2406.10260 [hep-ph]

[28] S. Alekhin, J. Blümlein, S. Moch, R. Placakyte, Parton distribution functions, , and heavy-quark masses for LHC Run II. Phys. Rev. D **96**(1), 014011 (2017). 10.1103/PhysRevD.96.014011. arXiv:1701.05838 [hep-ph]

[29] M.N. Costantini, L. Mantani, J.M. Moore, M. Ubiali, *A Linear PDF Model for Bayesian Inference* (2025). arXiv:2507.16913 hep-ph

